# Automated location invariant animal detection in camera trap images using publicly available data sources

**DOI:** 10.1002/ece3.7344

**Published:** 2021-03-10

**Authors:** Andrew Shepley, Greg Falzon, Paul Meek, Paul Kwan

**Affiliations:** ^1^ School of Science and Technology University of New England Armidale NSW Australia; ^2^ College of Science and Engineering Flinders University Adelaide SA Australia; ^3^ Vertebrate Pest Research Unit NSW Department of Primary Industries Coffs Harbour NSW Australia; ^4^ School of Environmental and Rural Science University of New England Armidale NSW Australia; ^5^ School of IT and Engineering Melbourne Institute of Technology Melbourne Vic. Australia

**Keywords:** animal identification, artificial intelligence, camera trap images, camera trapping, deep convolutional neural networks, deep learning, infusion, location invariance, wildlife ecology, wildlife monitoring

## Abstract

1. A time‐consuming challenge faced by camera trap practitioners is the extraction of meaningful data from images to inform ecological management. An increasingly popular solution is automated image classification software. However, most solutions are not sufficiently robust to be deployed on a large scale due to lack of location invariance when transferring models between sites. This prevents optimal use of ecological data resulting in significant expenditure of time and resources to annotate and retrain deep learning models.
2. We present a method ecologists can use to develop optimized location invariant camera trap object detectors by (a) evaluating publicly available image datasets characterized by high intradataset variability in training deep learning models for camera trap object detection and (b) using small subsets of camera trap images to optimize models for high accuracy domain‐specific applications.
3. We collected and annotated three datasets of images of striped hyena, rhinoceros, and pigs, from the image‐sharing websites FlickR and iNaturalist (FiN), to train three object detection models. We compared the performance of these models to that of three models trained on the Wildlife Conservation Society and Camera CATalogue datasets, when tested on out‐of‐sample Snapshot Serengeti datasets. We then increased FiN model robustness by infusing small subsets of camera trap images into training.
4. In all experiments, the mean Average Precision (mAP) of the FiN trained models was significantly higher (82.33%–88.59%) than that achieved by the models trained only on camera trap datasets (38.5%–66.74%). Infusion further improved mAP by 1.78%–32.08%.
5. Ecologists can use FiN images for training deep learning object detection solutions for camera trap image processing to develop location invariant, robust, out‐of‐the‐box software. Models can be further optimized by infusion of 5%–10% camera trap images into training data. This would allow AI technologies to be deployed on a large scale in ecological applications. Datasets and code related to this study are open source and available on this repository: https://doi.org/10.5061/dryad.1c59zw3tx.

## Graphical abstract

Achieve location invariant camera trap object detectors by using publicly available image data. Optimize object detectors for domain‐specific application using infusion of small subsets of camera trap images.

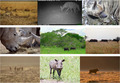

## 1 INTRODUCTION

Automated survey methods such as camera trapping and passive acoustic monitoring are widely used in ecological research (Gibb et al., 2019; Rovero & Zimmermann, 2016; Sugai et al., 2018). These methods provide invaluable insight into a plethora of ecological information including species occurrence, activity patterns, and behavior (O'Connell et al., 2011). However, they often result in the collection of large quantities of data, which must be processed, requiring a significant commitment of time and resources for manual or supervised classification (Swinnen et al., 2014; Young et al., 2018). Reducing the processing time and resources necessary for traditional data analysis such as manual analysis and citizen science (Nguyen et al., 2017; Swanson et al., 2015) has prompted increasing research into the adoption of Artificial Intelligence (AI) software in automated data classification (Falzon et al., 2014; Norouzzadeh et al., 2018; Willi et al., 2018).

Object detector and image classifier models have already been adopted to some extent in the processing of camera trap images (Falzon et al., 2020; Gomez Villa et al., 2016; Norouzzadeh et al., 2018; Tabak et al., 2019; Willi et al., 2018; Yu et al., 2013). These tools rely on data‐driven deep learning to identify complex patterns which can be used for classification without feature engineering as described by (Miao et al., 2019). However, most solutions presented thus far have shown limited transferability to image data outside the domain of the training data (Beery et al., 2018; Willi et al., 2018). This results in the need to develop models specific to each domain. However, this process is time and resource intensive, requiring repeated collection and manual annotation of camera trap data, and computationally expensive training of deep neural networks (Falzon et al., 2020). Thus, there is a clear need to develop location invariant object detectors, which are deep learning models that can be transferred from one location to another, achieving acceptable results without having to be retrained. Such out‐of‐the‐box solutions are attractive due to their potential for extensive application, particularly in circumstances where the development of domain or study‐specific models is prohibitively expensive or otherwise unattainable.

Achieving location invariance requires training data to be characterized by high intradataset variability. This is because neural networks learn patterns in data, meaning low intradataset variability can result in learning of domain‐specific features such as camera angle, lighting, and vegetation, reducing location invariance (Miao et al., 2019; Singh et al., 2020; Torralba & Sinha, 2003). Therefore, camera trap images must be obtained from many sources to be able to train effective object detectors and classifiers. This requires the deployment of many camera traps across large geographical regions and environments. However, establishing such extensive networks of cameras is time and resource intensive and may be unfeasible for smaller‐scale studies or those focusing on rare or elusive species. Even when researchers have access to a network of camera traps, collecting enough images for training object detectors can prove difficult. (Maurice, 2019) deployed 15 cameras for 2 months resulting in the collection of only 41 images of the pangolin (the target species), a number which would be insufficient for effective neural network training (Shahinfar et al., 2020). Other factors which limit the accessibility and availability of camera trap images include the reticence of researchers to share existing camera trap data, or lack of data for novel species studies.

These limitations in data accessibility and availability limit the adoption of automated AI solutions in ecological camera trap image processing (Schneider et al. 2018). Thus, alternative data sources must be identified and evaluated to assist in the development of object detectors capable of being deployed in any domain, at any location, achieving acceptable results regardless of camera trap image availability. Possible solutions include publicly available sources of animal imagery, such as FlickR (flickr.com) and iNaturalist (inaturalist.org). FlickR is a consumer photo sharing website, hosting approximately 10 billion images, shared by over 90 million monthly users. It is characterized by high intradataset variability, high accessibility, and a wide range of species types in highly varying contexts, with minimal unintentional bias, as images are not collected for a specific purpose (Everingham et al., 2010). It is arguably the most extensively used source of image data in object detection benchmark datasets, including ImageNet (Deng et al., 2009), MS COCO (Lin et al., 2014), the Open Images Dataset (Kuznetsova et al., 2020) and PASCAL VOC (Everingham et al., 2010). iNaturalist contains over 45 million observations of biodiversity data including both flora and fauna. Labeling of images on iNaturalist may be more accurate than FlickR due to its purpose as a biodiversity data sharing website, and it does contain more camera trap images than FlickR. Other potential image sources include Pinterest (www.pinterest.com), Imgur (www.imgur.com), pixabay (www.pixabay.com), and 500px (www.web.500px.com). These image sources are highly beneficial in training general, location invariant neural networks as they exhibit an extensive range of contextual features, not necessarily present in camera trap imagery.

Despite their benefits as out‐of‐the‐box solutions, universal or general object detectors usually fail to achieve the high accuracy attainable by domain‐specific object detectors (Rebuffi et al., 2017; Wang et al., 2019). Due to the need to achieve high accuracy object detection and classification in ecological research, it may therefore be necessary to optimize location invariant models for domain‐specific studies. This is particularly relevant when processing camera trap imagery characterized by features which differ strongly from noncamera trap data, including infrared imagery, poor‐quality illumination and blurry images.

- To evaluate the use of publicly available image sources, in the development of location invariant camera trap object detectors.
- To develop an optimization strategy dubbed ‘infusion’ to improve the performance of location invariant object detectors in domain‐specific applications.

Therefore, the aims of this study are twofold:

In this study, we will demonstrate our proposed approach on three single‐class applications. The rare species Striped Hyena (*Hyaena hyaena*) was chosen due to the sparsity of camera trap training data, and the difficulty in discriminating between the striped hyena and the more common spotted hyena. Furthermore, other studies have highlighted it as a species of particular interest due to the difficulty they faced in detecting its presence in camera trap images, for example, (Willi et al., 2018) failed to detect any of the 27 striped hyenas present in their test dataset. Next, the iconic and critically endangered Rhinoceros (*Rhinocerotidae*) was also chosen, due to the high research interest in monitoring its prevalence and changes in populations. Finally, the pest family *Suidae* (pigs, boars and hogs) was included due to the significant role it plays across global ecosystems and its host status for a range of diseases such as Swine Fever, which are a major threat to agricultural industries.

## 2 METHODOLOGY

### 2.1 Datasets and annotation

The datasets used in this study were collated using images from FlickR and iNaturalist. We also used camera trap image datasets obtained from www.lila.science including Snapshot Serengeti (SS), Wildlife Conservation Society (WCS) Camera Traps, and other sites specified in more detail below. All datasets, annotations, and the algorithms used for dataset collection and processing, as well as auto‐annotation of images are available at: https://doi.org/10.5061/dryad.1c59zw3tx.

#### 2.1.1 FlickR and iNaturalist

We developed and used a Python script to download images from FlickR using the FlickR API. This allowed us to download images with multiple keywords at once. The keywords used are shown in Table 1. We downloaded a maximum of 200 images per keyword, to maximize the variety of search results. Our datasets were restricted to Creative Commons images. We also developed a Python script to download images from iNaturalist using a csv file containing URLs of relevant observations downloaded from inaturalist.org.

**TABLE 1:** Keyword searches used to download images from FlickR and iNaturalist. Scientific names tended to return more accurately labeled images

Rhinocerotidae	Hyaena hyaena	Suidae
*diceros AND bicornis* *ceratotherium AND simum* *dicerorhinus AND sumatrensis* *white AND rhinoceros* *rhinoceros*	*striped AND hyena* *Hyaena AND hyaena*	*Phacochoerus AND africanus* *Sus AND scrofa* *sanglier* *warthog OR warthogs* *wild AND pig OR boar OR hog* *feral AND pig OR boar OR hog*

**FIGURE 1:**
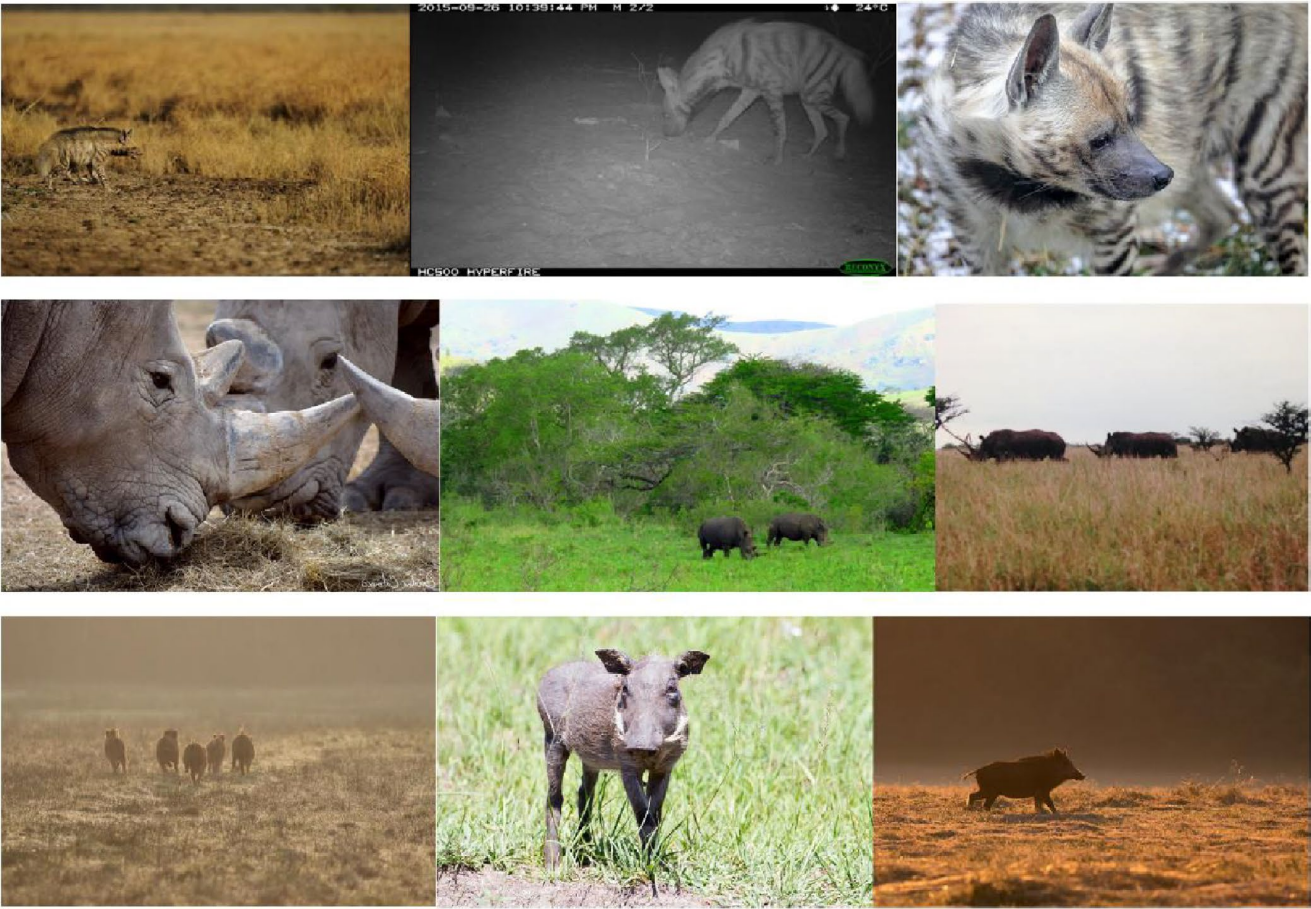
Subsamples of the FiN datasets. Top to bottom: striped hyena, rhinoceros, and pig. Images of were highly varied, and included both color/daytime and infrared images, as well as a large range of contexts and distances from the camera

Duplicates and near duplicates were removed using a Structural Similarity Index (SSIM; Zhou et al., 2004) clustering algorithm we developed (see Appendix S4). We deleted all images with a similarity score above 0.8, where a score of 1.0 represents a 100% similarity between 2 images. Near duplicates are images with strong visual similarity, containing only small distortions, slight variations, and occlusions (Everingham et al., 2010). Interestingly, the datasets downloaded from FlickR and iNaturalist were mutually exclusive, with not one image present on one site, being also present on the other. Although this does not mean that images obtained from FlickR will not be available via iNaturalist, it does suggest that users of FlickR may often not be users of iNaturalist. Details about the final datasets are shown in Table 2. Subsamples of the final datasets are illustrated by Figure 1.

**TABLE 2:** Final number of images obtained from FlickR and iNaturalist for both the single‐class and multi‐class experiments, after duplicate removal and cleaning. Datasets are referred to hereon according to their source, abbreviated as FiN (FlickR–iNaturalist) and class name

Dataset name	Class	FlickR	iNaturalist	Total images
FiN_rhino	*Rhino*	784	881	1,665
FiN_striped_hyena	*Striped hyena*	401	71	472
FiN_pig	*Pig*	606	0	606

#### 2.1.2 Camera trap datasets

We obtained all camera trap data of rhinoceros and striped hyena from lila.science using a Python script we developed, which we have made available on our Dryad repository. We scoured all images of striped hyena and rhinoceros from both WCS Camera Traps (*WCS_striped_hyena* and *WCS_rhino*) and Snapshot Serengeti (*SS_striped_hyena* and *SS_rhino*) datasets (Swanson et al., 2015). We used the same script to obtain our *EU_pig* and *NA_pig* datasets from the Missouri Camera Traps (Zhang et al., 2016) and North American Camera Trap Images (Tabak et al., 2018) datasets, respectively, also from lila.science. A summary of all camera trap datasets is provided in Table 3. Note in all experiments, the out‐of‐sample test sets are comprised of the Snapshot Serengeti datasets.

**TABLE 3:** Summary of the characteristics of the camera trap datasets used in this study. The term “quality” refers to characteristics such as blurriness, pixilation, illumination etc. A poor‐quality dataset will contain many images that are over or underexposed, blurriness caused by poor focus, or other features which make it harder to distinguish the identity of a target class and distort or damage key features. A visual subsample of these datasets is provided (see Figure 2)

Dataset	Source	Location	Size	Characteristics
*WCS_striped_hyena*	Wildlife Conservation Society	Multiple	582	Moderate quality Night and day
*SS_striped_hyena*	Snapshot Serengeti	Tanzania	478	Moderate quality Infrared and day Includes partials
*WCS_rhino*	Wildlife Conservation Society	Multiple	333	Low quality Mostly infrared Many partials
*SS_rhino*	Snapshot Serengeti	Tanzania	153	Moderate quality Daytime Many partials
*AU_pig*	Custom	NSW, Australia	589	Low quality Mostly infrared High occlusion High density
*SS_pig*	Snapshot Serengeti	Tanzania	574	Moderate quality Mostly daytime
*CC_pig*	Camera CATalogue	South Africa	559	Moderate quality Partials Low density
*NA_pig*	North America Camera Trap Images	United States	514	High quality
*EU_pig*	Missouri Camera Traps	Europe	501	Difficult High occlusion

**FIGURE 2:**
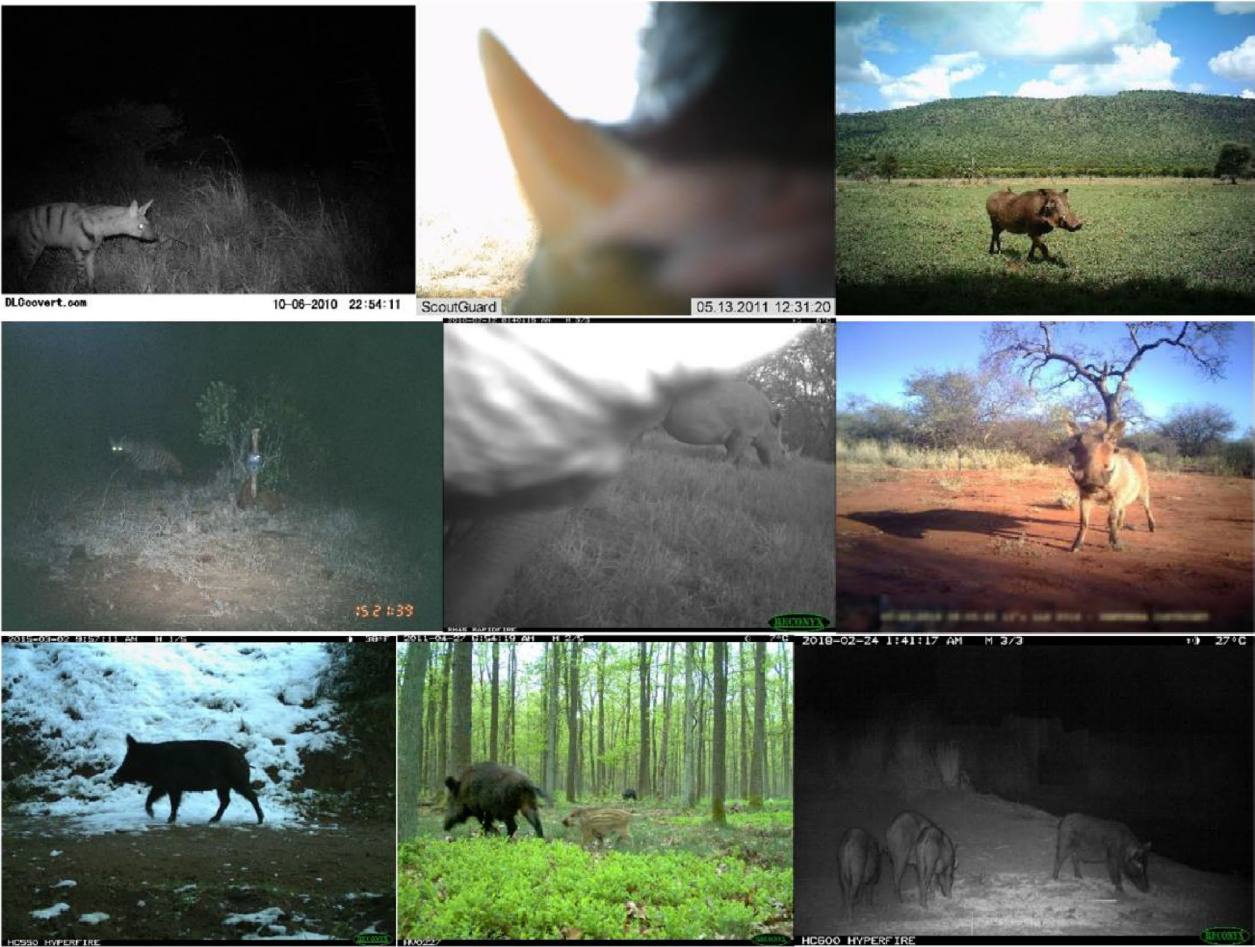
Subsamples of the camera trap datasets. Top row: SS datasets, left to right; striped hyena, rhino, and pig. Middle row: left; WCS_striped_hyena, middle; WCS_rhino, right; SS_pig. Bottom row: left; NA_pig, middle; EU_pig and right; AU_pig

The *SS_pig* dataset is a subset of the Snapshot Serengeti dataset, and *CC_pig* is a subset of the Camera CATalogue project conducted by Panthera (www.panthera.org). Both are available from the Data Repository for the University of Minnesota, used by (Willi et al., 2018), and released under a CC0 1.0 Universal Public Domain Dedication license. The Australian pig dataset (AU_pig) is a custom dataset, obtained during feral pig trapping and control operations. More information about each dataset is provided in Table 3, and a subset is shown in Figure 2.

Each image in the final datasets was annotated with bounding boxes and corresponding class labels. Bounding box annotation involves the positioning of an axis aligned box surrounding an object. We used an auto‐annotator tool we developed to roughly annotate all the images. We then edited any suboptimal bounding boxes using the graphical annotation tool labelImg (Tzutalin 2015; https://github.com/tzutalin/labelImg) to ensure all objects were correctly annotated. Annotations were saved in PASCAL VOC format.

### 2.2 Training and evaluation methodology

In this study, we conducted two major experiments. First, we compared the performance of models trained on FlickR–iNaturalist (FiN) datasets only to those trained only on camera trap data using evaluation on out‐of‐sample test sets. Next, we optimized the FiN models by infusing small subsets of camera trap imagery into the FiN training set, evaluating performance on out‐of‐sample test sets. Details about the model architecture and training parameters are provided in Appendix S3. Additional information on transfer learning is also provided. The experiments outlined in this section were also verified on a multi‐class application documented in Appendix S5.

#### 2.2.1 Comparison between FiN and camera trap data in developing location invariant object detectors

To evaluate the potential for publicly available data from FlickR and iNaturalist to be used in the development of location invariant object detectors for camera trap image processing, we trained Keras‐RetinaNet (Lin et al., 2018) models on FiN datasets, and compared their performance to that of RetinaNet models trained on camera trap data when tested on out‐of‐sample camera trap images.

We trained three single‐class RetinaNet models on FiN datasets. These models are referred to as *FiN_Classname*; for example, *FiN_rhino* refers to a rhino detector trained on FiN data. We also trained two single‐class (rhino and striped hyena) RetinaNet models using the *WCS_striped_hyena* and *WCS_rhino* datasets, as well as four pig detectors, on the *AU_pig*, *CC_pig*, *NA_pig,* and *EU_pig* datasets. All models are named based on the source of their training data. Note, we were able to train four pig models due to greater availability of data when compared with rare species such as rhino and striped hyena.

The datasets were randomly split into training and validation sets, with 90% of images reserved for training, and 10% used for validation. Each training set was supplemented with 800 explicit negative samples to improve discrimination between target species and nontarget species or background. A detailed breakdown of the training and validation splits as well as the out‐of‐sample test set is provided in Table 4.

**TABLE 4:** Data distribution for models trained on datasets obtained from FlickR/iNaturalist, abbreviated as FiN (FlickR–iNaturalist), and models trained using camera trap images alone abbreviated as follows; WCS (Wildlife Conservation Society), AU (Australia), NA (North America), CC (Camera CATalogue), and EU (Europe). All models were tested on out‐of‐sample images obtained from Snapshot Serengeti

Models	Training set (90%)	Validation set (10%)	Out‐of‐sample test set (SS)
*FiN_striped_hyena*	425	47	478
*WCS_striped_hyena*	524	58	
*FiN_rhino*	1,499	166	153
*WCS_rhino*	300	33	
*FiN_pig*	545	61	574
*AU_pig*	530	59	
*CC_pig*	503	56	
*NA_pig*	463	51	
*EU_pig*	451	50	

All models were tested using out‐of‐sample images from the Snapshot Serengeti (SS) datasets, that is, *SS_striped_hyena*, *SS_rhino*, and *SS_pig*. Each test set was supplemented with 200 negative samples to prevent biased evaluation of false positives. These negative samples were derived from the Snapshot Serengeti, and consisted of empty images, or images of nontarget species. For more information relating to the negative sampling data collection process, refer to Appendix S2.

#### 2.2.2 Infusion: Optimization of location invariant models using camera trap imagery

Next, we conducted experiments to evaluate an optimization process that would allow ecologists to improve object detection performance with minimal infusion of camera trap images into the FiN training set. Infusion is the process of supplementing the training set with a small subset of camera trap images, to improve robustness to the particularities of camera trap data, such as infrared, high occlusion, and blurriness. Infusion was conducted both out of sample and in‐sample. Out‐of‐sample results are presented in this manuscript. For in‐sample results, refer to Appendix S6.

Due to the large number of highly similar images present within camera trap datasets, the infusion subsets were not randomly selected. Instead, our SSIM algorithm was used to retain only images with low SSIM scores, with the aim of maximizing intradataset variability. The SSIM algorithm allowed us to randomly select one frame from each cluster of images (usually one capture event, or different capture events with very similar properties).

**FIGURE 3:**
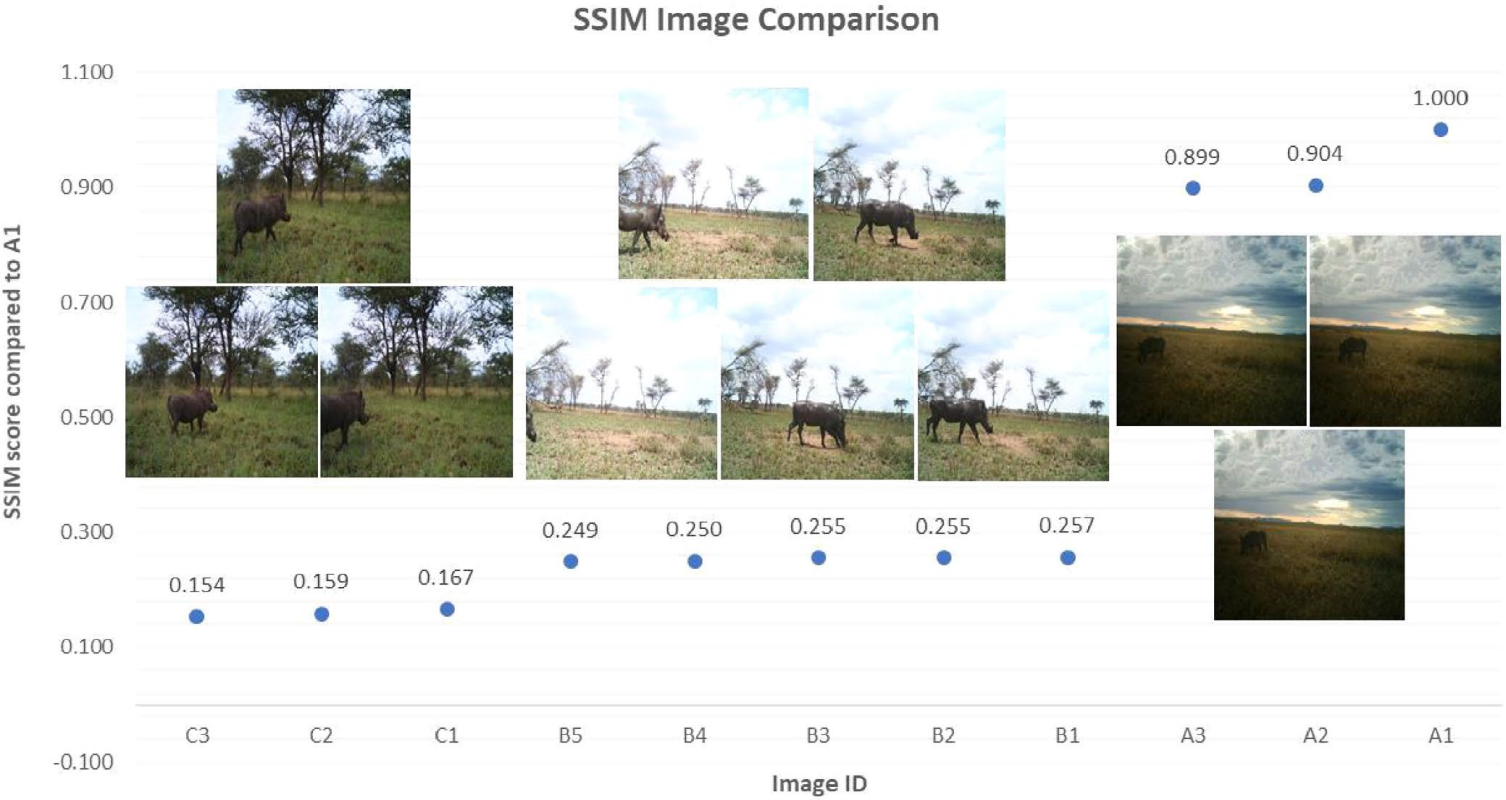
Graphical illustration of image clustering using an SSIM algorithm. The test image represented by 1.0 is compared with every other image. Highly dissimilar images have low SSIM scores (<0.4)

Our research indicates that image pairs with an SSIM value above 0.4 have sufficiently high similarity to be clustered. For example, Figure 3 illustrates the output of the SSIM algorithm graphically, clearly showing the three clusters formed by visually similar images; the image in the upper right section of the graph (A1) is compared to each other image, with values closest to 1 indicating high similarity with the test image. This method allows researchers to compile highly varied datasets automatically, minimizing the need for extensive time‐consuming image sorting and annotation.

Out‐of‐sample infusion was conducted by training four additional models for each species, with incremental infusion of the SSIM sorted camera trap images from the WCS and CC datasets into the FiN training data. These images were added in increments of 5% from 5% to 20%, as shown by Table 5. For example, the *FiN_rhino* dataset comprised of 1665 images. To achieve 5% infusion, 83 images from the *WCS_rhino* dataset were added to the *FiN_rhino* dataset. 90% of these images were retained for training, with 10% reserved for monitoring training via the validation set. This process was repeated for all percentages and species shown in Table 5.

**TABLE 5:** Incremental infusion of camera trap images into FiN training. The additional 800 negative samples were included in the training set. Models are named according to the class name and infusion percentile. Note the infusion images are trap images. The infusion training set is made up of FiN+ infusion images. The validation set is FiN validation+ infusion images

Class	Model name	Infusion Source	No infusion images	Infusion training set	Infusion Validation set
Hyaena	hyaena_inf_05	WCS_hyena	24	446	50
	hyaena_inf_10		47	467	52
	hyaena_inf_15		71	489	54
	hyaena_inf_20		94	509	57
Rhino	rhino_inf_05	WCS_rhino	83	1573	175
	rhino_inf_10		167	1649	183
	rhino_inf_15		250	1723	192
	rhino_inf_20		333	1798	200
Pig	pig_inf_05	CC_pig	30	572	64
	pig_inf_10		61	600	67
	pig_inf_15		91	627	70
	pig_inf_20		121	654	73

The models were then tested on the out‐of‐sample Snapshot Serengeti test sets presented in Table 4. Both the training and test sets were supplemented with negative samples as described in Section 2.2.1.

#### 2.2.3 Model evaluation

To evaluate the performance of our models, mean Average Precision (mAP) results will be provided. mAP is calculated as documented in the PASCAL VOC benchmark (Everingham et al., 2010). A high mAP indicates that the model is detecting the majority of objects with high accuracy, and minimal retention of false positives. Accuracy is measured using Intersection over Union (IoU), which is a measure of the overlap between the detection box and the ground truth bounding box.

We also evaluate the performance of our infusion models at varying confidence thresholds. A confidence threshold is preset by users of object detectors to ignore low scoring detections. When an object detector locates features of a target class, it attributes a classification score to the region of interest. If the classification score is low, it can be excluded using a high confidence threshold. This allows more confident detections to be retained while reducing false positives.

## 3 RESULTS

### 3.1 Comparison between FiN and camera trap data in developing location invariant object detectors

**FIGURE 4:**
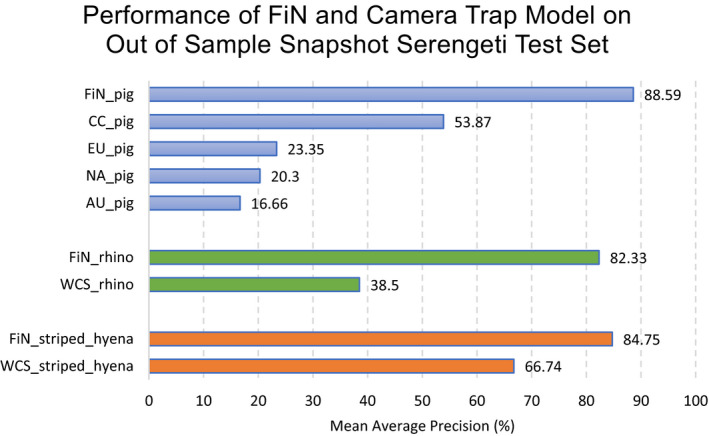
Comparison of the mAP results achieved by the models trained on FiN data, and those trained on camera trap datasets. In all cases, the FiN models outperformed the camera trap models

The results of training on FiN data compared with training on camera trap data are presented in Figure 4. All results were collected on the out‐of‐sample Snapshot Serengeti test sets. The models trained on FiN datasets achieved mAP results ranging between 82.33% and 88.59%, while the models trained on camera trap data achieved mAP results ranging from 38.5% to 66.74%. In all cases, the FiN models outperformed the models trained on camera trap images.

The *FiN_pig* model achieved a mAP of 88.59% when tested on the out‐of‐sample *SS_pig* dataset. This was far superior to the *CC_pig* model, which was trained on camera trap images of warthogs from the Camera CATalogue (CC) dataset, achieving a mAP of only 53.87%. Although both the *CC_pig* dataset and the *SS_pig* dataset contained the same subspecies (*Phacochoerus africanus*), the *CC_pig* model did not generalize well to the *SS_pig* test set. This may be because the *SS_pig* dataset was characterized by more variation in background, greater variation in the distance of pigs from the camera and greater contrast. Notably, the worst performing pig model was trained on data from Australia (*AU_pig*). This is very likely due to the large number of low quality infrared images present in the training data, which caused the model to return a high rate of false positives, and the large disparity between contextual features such as vegetation and species type (the Australia subspecies was *Sus scrofa*, while the SS subspecies was *Phacochoerus africanus*).

In comparison, the significantly greater intradataset variability present in the FiN datasets allowed for better model generalization when compared to the models trained only on single location camera trap data. This trend was observed across all classes, with the *FiN_striped_hyena* and *FiN_rhino* models significantly outperforming the *WCS_striped_hyena* and *WCS_rhino* models.

### 3.2 Infusion: Optimization of location invariant models using camera trap imagery

**FIGURE 5:**
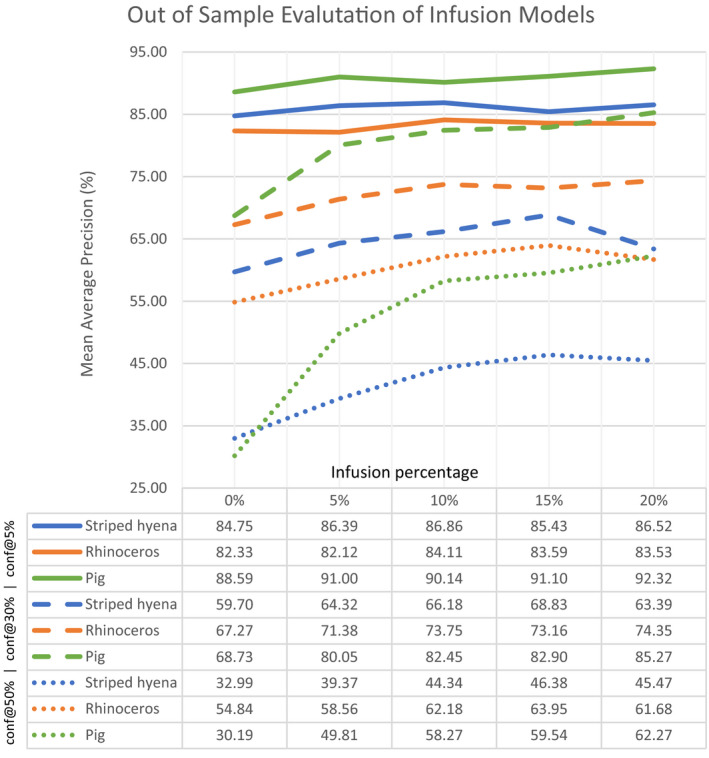
Results of the infusion experiments on the out‐of‐sample SS test set. Infusion resulted in improvement across all models, particularly when evaluated at higher confidence thresholds. Infusion of 5% significantly improves performance; however, optimum performance occurs at 10%–15%, with the mAP results plateauing beyond 15%

The results presented in the previous section indicate that the models trained on FiN datasets can be used to effectively process images collected at any camera trap site with an acceptable level of location invariance. However, camera trap images possess particular characteristics which differentiate them from FiN images. In difficult cases, the mAP achieved by FiN models may not be sufficiently high for practical purposes, particularly when higher confidence thresholds are used. For example, for a given study, the confidence threshold may be set to 50%, meaning all detections with a classification score lower than 50% would be ignored. Thus, we present the results of our infusion optimization experiments, illustrated by Figure 5. In all cases, infusion resulted in an increase in mAP when evaluated on out‐of‐sample images.

At a confidence threshold of 5% (the standard threshold for mAP measurement (Lin et al., 2018)), out‐of‐sample infusion did not result in a pronounced improvement, with gains in mAP results ranging from 1.78% to 3.73%. However, in practical deployment, a confidence threshold of 5% would rarely be used, with ecologists favoring higher thresholds to ensure confident classification of species. It is at these higher thresholds that the benefits of infusion are best demonstrated. For example, at a confidence threshold of 30%, the mAP improved by 7.08%–16.54%, while at a confidence threshold of 50% it improved by 9.11%–32.08%.

The results presented in Figure 5 indicate that the addition of a small percentage of camera trap images into the FiN training dataset can significantly improve performance. In most cases, the greatest improvement occurred with infusion of 5%, with performance continuing to improve as infusion was increased to 15%. As infusion was increased beyond 15%, performance plateaued, or decreased, with only 4 out of 9 results improving beyond 15%.

In some cases, in‐sample images may be necessary to boost performance further, particularly in circumstances where domain‐specific images contain unusual features not present in FiN or out‐of‐sample infusion data. As such, we present results of in‐sample infusion experiments in Appendix S6. In all cases, 5%–10% in‐sample infusion resulted in significant gains in mAP (3.66%–18.20%). Further infusion provided some accuracy gains (1.41%–4.12%). These results collected with a confidence threshold of 5% can be compared to the gains of 1.78%–3.73% gained by out‐of‐sample infusion with a 5% confidence threshold. However, it must be noted that in‐sample infusion also tends to result in greater retention of false positives which may damage mAP results at higher confidence thresholds. For further discussion, see Appendix S6.

## 4 DISCUSSION

We investigated the use of FiN images as an alternative to camera trap images in the task of DCNN training for location invariant camera trap image processing tasks, on three case studies, namely, striped hyena, rhinoceros, and pig. Specifically, we established the greater transferability of the FiN trained models when compared to models trained on camera trap datasets, and their high usability as location invariant object detectors. We then demonstrated how such models can be optimized via out‐of‐sample infusion, which was shown to increase the confidence of detections, allowing more true positives to be retained at higher confidence thresholds.

Our results show that FiN training significantly improves model robustness and location invariance. Particularly, it provides ecologists with a practical, cost‐effective, out‐of‐the‐box solution, capable of detecting animals even in the most challenging camera trap environments. We not only established that FiN data alone can be used to achieve good results, but these models can be improved with 5%–10% infusion of out‐of‐sample or in‐sample camera trap data to improve robustness to the particularities of camera trap imagery. One limitation of this study is that it only evaluates the models in terms of the Snapshot Serengeti dataset. We could only evaluate on one dataset for the classes “striped hyena” and “rhinoceros” due to lack of data availability. To maintain consistency, we also only presented results for the class “pig” on Snapshot Serengeti in this manuscript. However, to verify the usability of this method at any location and for any dataset, we present more extensive results in Appendix S7 for the class pig, for which we had more data available, thus showing location invariance across four extra test locations.

Out‐of‐sample infusion was demonstrated to significantly improve the classification scores attributed to positive detections, thus allowing them to be retained even when using a higher confidence threshold. It is well established that increasing the confidence threshold decreases recall (the number of true positives retained in the final output), and consequently decreases mAP (Willi et al., 2018). Note, we did not conduct evaluations of the models at confidence thresholds above 50% because almost all detections with scores above 50% were true positives, which meant increasing the threshold simply removed true positives. Selecting a confidence threshold for a given application is highly dependent on the quality of training data, extent of negative sampling and the model used (Beery et al., 2018). The supplementation of FiN training with out‐of‐sample camera trap imagery is therefore highly beneficial as it allows more true positives to be retained, because the overall confidence of correctly detected objects is improved. This is a result of the improved robustness to the particularities of camera trap imagery.

Our results suggest that ecologists can train object detectors using FiN imagery, and if camera trap data are available for their target species, use it to infuse the FiN training data. This model can then be used to process out‐of‐sample images from any camera trap, achieving a sufficiently high mAP to be deployed in most applications (Glover‐Kapfer et al., 2019; Wearn & Glover‐Kapfer, 2017). Furthermore, in circumstances where model performance is still considered suboptimal, they may then infuse model training with in‐sample camera trap images, for further optimization. Although in‐sample infusion makes the model more location variant, it does provide a means by which ecologists can train powerful models capable of achieving mAP results above 90%, with very few training images, as demonstrated by the results of in‐sample infusion presented in Appendix S6. As shown by various studies in automated camera trap image processing, achieving robust object detectors via training solely on camera trap images usually requires thousands to millions of images (Norouzzadeh et al., 2017; Tabak et al., 2019; Willi et al., 2018). In‐sample infusion overcomes this requirement by leveraging off the robustness of the FiN model, and the strong availability of FiN imagery to allow ecologists to train high accuracy optimized deep leaning models with very few camera trap images, significantly reducing the time and resources necessary to develop automated deep leaning object detectors.

In light of the growing number of camera trap‐based projects undertaken by ecologists (Christin et al., 2019; Glover‐Kapfer et al., 2019; O’Connell et al., 2011; Rovero & Zimmermann, 2016), this research provides an invaluable method by which researchers can process extensive image data regardless of the location from which the images were obtained, and the particularities of the camera trap site or species. This method has been proven on several species, including rare species, for which camera trap data for training models is often sparse. As illustrated by (Willi et al., 2018), the lack of camera trap data for rare species poses significant problems when training multi‐class object detectors, as the large class imbalance between common species and rare species causes object detectors to misclassify species, by over enthusiastically classifying species based on how common they are in the dataset rather than via their features. This was observed by (Willi et al., 2018) who noted that insufficient images of the rare striped hyena in their dataset resulted in their model achieving a mAP of 0% on this class. We have specifically addressed this problem by proposing the use of FiN images of striped hyena to rectify limitations in data availability.

The use of FlickR as the principal training data also rectifies another major problem faced by researchers. Studies have indicated that deep learning models have a tendency to return overly confident predictions (Beery et al., 2018; Meek et al., 2015; Schneider et al., 2019; Willi et al., 2018) when trained on camera trap data and deployed in‐sample. This is due to the high consistency in image quality, lighting, camera angle, and geographical and vegetation features in camera trap data (Everingham et al., 2010). Furthermore, many trap images feature obscured or poor‐quality imagery of animals which if used in the training set, may cause the network to make unrealistically optimistic predictions, by attributing 100% confidence to visual features which may not display sufficiently distinct characteristics present solely in the target class (Ponce et al., 2007). In contrast, the higher resolution of FiN images and large variations between images forces the model to reduce the confidence attributed to poor‐quality or obscured animals. Their greater robustness allows them to be deployed out of sample, further minimizing this problem.

One potential benefit in using FiN imagery for training image processing models is the high availability of already annotated animal images. Because FlickR is a major source of images used in datasets such as ImageNet (Deng et al., 2009) and MS COCO (Lin et al., 2014), many animal classes have already been annotated with bounding boxes, which are freely available for downloading. Using the method proposed in the paper would therefore significantly reduce the time and resource expenditure necessary for model development, by leveraging off the work already completed by the broader object detection community. We were unable to use annotated FlickR images from ImageNet as it was under maintenance; however, it may prove to be a valuable resource in the development of future models. This study was limited to the evaluation of FlickR and iNaturalist images, and did not evaluate alternative images sources mentioned in Section 1.

This research did not investigate the application of the FiN and infusion training method using alternative object detectors such as YOLO (Redmon & Farhadi, 2016), and Faster R‐CNN (Ren et al., 2015). Applying the findings of this study to these architectures may be beneficial. YOLO is a faster, more efficient object detector, which may be more suited to video processing, while Faster R‐CNN generally achieves higher accuracies, but is slower. RetinaNet was chosen as it achieves a good balance between the computational efficiency of YOLO and the accuracy of Faster R‐CNN, which made it an appropriate choice for the difficult task of camera trap image processing (Yang et al., 2019). In this study, we have only demonstrated location invariance using RetinaNet. Although it goes beyond the scope of this study, it would be interesting to ascertain whether changes in model architecture would influence the robustness of location invariance models. Another possible area of research could be the application of this method to object segmentation‐based image processing. Object segmentation builds upon the benefits of object detection by excluding background features. This limits the influence of contextual features on model performance, thus improving model accuracy and overall performance; however, it is likely that they would encounter the same modeling bias faced by bounding box‐based object detection models.

Finally, the proposed method may be extended to other image modalities. For example, it could be extended to drone imagery (Kellenberger et al., 2017; Xu et al., 2020). Drone images are often captured from an aerial perspective, meaning they would contain quite different features to those present available on FlickR. Applying our findings to object detection in the context of drone imagery would be interesting, particularly with infusion of a small subset of drone images to boost performance and allow better generalization to the particularities of drone imagery. This would determine how transferable FiN images are to new modalities. It could also be extended to other applications such as underwater animal imagery (Christensen et al., 2018; Dawkins et al., 2017), surveillance footage (Raghunandan et al., 2018), and thermal camera imagery (Bondi et al., 2020; Rodin et al., 2018). This may present opportunities to rectify image shortages, or problems with low intradataset variability, particularly in novel studies.

## 5 CONCLUSION

This study successfully demonstrated the use of FiN datasets in training location invariant deep learning object detection models in the task of camera trap image processing. It also evaluated an optimization process dubbed infusion, to improve robustness to the particularities of camera trap imagery. Results presented across three single‐class models on out‐of‐sample test sets indicate the aims of this study have been achieved. However, our approach is limited by its inability to achieve high precision out‐of‐sample object detection, which is still best achieved via in‐sample training or infusion. Furthermore, this method was not evaluated on alternative object detection frameworks and did not provide findings on an extensive multi‐class dataset. Nevertheless, this study provides a promising pathway to develop robust, location invariant models using publicly accessible data sources. Furthermore, development of these models will facilitate the widespread deployment of AI in ecological management. The findings of this study could also be extended beyond camera trapping to other object detection tasks and image modalities such as drone imagery. Furthermore, the methodology of using transfer learning and publicly available datasets characterized by high intradataset variability and minimal unintentional bias to train location and context invariant AI‐based data processing software could be extended beyond images to other forms of data.

## CONFLICT OF INTEREST

None to declare.

## AUTHOR CONTRIBUTION

**Andrew Jason Shepley:** Conceptualization (lead); Data curation (lead); Formal analysis (lead); Investigation (lead); Methodology (lead); Resources (equal); Validation (equal); Visualization (lead); Writing‐original draft (lead); Writing‐review & editing (lead). **Greg Falzon:** Conceptualization (equal); Formal analysis (equal); Funding acquisition (lead); Project administration (equal); Resources (equal); Supervision (equal); Writing‐review & editing (equal). **Paul D. Meek:** Funding acquisition (equal); Resources (equal); Writing‐review & editing (equal). **Paul Kwan:** Supervision (equal); Writing‐review & editing (equal).

## Supporting information

Appendix S1

Appendix S2

Appendix S3

Appendix S4

Appendix S5

Appendix S6

Appendix S7

## Data Availability

*Image and Annotation datasets:* All image datasets and corresponding annotations are available on Dryad (https://doi.org/10.5061/dryad.1c59zw3tx). *Code and scripts:* All code and scripts are available on Dryad (https://doi.org/10.5061/dryad.1c59zw3tx).
