# Supplementary material for: Automated location invariant animal detection in camera trap images using publicly available data sources
Source: Ecol Evol. 2021 Mar 10;11(9):4494–506. doi: 10.1002/ece3.7344 (PMC8093655; doi:10.1002/ece3.7344)
Supplement: Supplementary file 1 — Appendix S1 [file ECE3-11-4494-s005.pdf]

APPENDIX S1 - Related Work

1. Traditional Methods: Manual Analysis and Citizen Science

The majority of camera trap image processing is achieved by manual analysis conducted by ecologists, or via citizen science. Manual analysis involves the use of software programs to manually tag animals in images/capture events. Each image sequence or capture event is treated as a detection, and the ecologist must manually select a tag reflecting the identity of the animal. Once tagging is complete, a verification process is undertaken to identify and correct mistaken classifications. These tagged images can then be interrogated according to the purpose of the study, using tools such as R scripts, or specially developed GUI programs. Manual analysis of images is a significant resource demand on ecologists and research teams, requiring large expenditures in time and resources, hindering effective biodiversity management.

This time-consuming task may also be undertaken by citizen scientists, who are volunteers that contribute to scientific enquiry by collecting or processing image data (Nguyen, Maclagan et al. 2017). Large citizen science-based programs such as Zooniverse (www.zooniverse.org) enable the effective classification of millions of camera trap images (Jones, Allen et al. 2018). Citizen science projects have many benefits for researchers including customization of projects and annotation requirements in accordance with the aims of projects. However, the effectiveness of citizen science in rapidly processing large volumes of image data with sufficient accuracy is limited (Meek and Zimmerman 2016), causing large delays between the data collection and interpretation stages, which may be detrimental to ecological management (Fox, Bourn et al. 2019). Furthermore, the need to upload significant

amounts of data onto publicly accessible websites may pose privacy risks (Sagarra, Gutiérrez-Roig et al. 2015) or poaching concerns and undermine the protection of rare or endangered species by revealing their geographical location and behavioral habits to poachers (Falzon, Lawson et al. 2020).

2. Automated Image Processing Using Deep Learning

Due to the shortcomings of traditional methods, research has centered primarily on integration of automated image processing within camera trap research (Meek, Fleming et al. 2014, Meek, Ballard et al. 2015, Fegraus and MacCarthy 2016, Willi, Pitman et al. 2018, Young, Rode-Margono et al. 2018). To achieve this, neural networks such as Deep Convolutional Neural Networks (DCNNs) are trained on large amounts of annotated image data (thousands to millions of images) to recognize discriminative features belonging to target classes (Zhao, Zheng et al. 2019). Handcrafted features specified by researchers are not used, instead the features are 'learned' via updating of weights during training. When the DCNN is confident in the presence of an object in an image, it maps bounding boxes, segmentation masks, or classification labels to the image or object (Ren, He et al. 2015). If a DCNN is very deep, consisting of many layers, it will have many trainable parameters (usually millions) which gives rise to the need for large annotated image datasets used in training these parameters from scratch. This is necessary for the network to learn complex features (Samala, Chan et al. 2016). Although DCNNs can be used to classify data with high accuracy, their usability can be limited by insufficient training data which may lead to overfitting (memorization of training data), and consequently, inability of the model to generalize to new data (Zhao 2017).

Early attempts at automated camera trap classification and object detection tasks using neural networks were dependent on significant amounts of pre-processing (Yu, Jiangping et al. 2013) and resulted in relatively poor accuracy (Swinnen, Reijniers et al. 2014, Chen, Han et al. 2015). However, most modern solutions use minimal pre-processing, or automate pre-processing (Giraldo Zuluaga, Salazar et al. 2017). Accuracy and recall attained by deep learning solutions is also increasing significantly, as large annotated datasets become available and progress is achieved in training methods, such as the adoption of transfer learning (Gomez Villa, Salazar et al. 2016, Willi, Pitman et al. 2018). Transfer learning involves the repurposing of learned features for another task (Yosinski, Clune et al. 2014). This allows general features learned on a large, highly varied dataset such as ImageNet (Deng, Dong et al. 2009) which contains 3.2 million images, or Snapshot Serengeti (Swanson, Kosmala et al. 2015), which contains 7.3 million images to be transferred to a smaller, similar dataset containing only hundreds to thousands of images. Transfer learning has been shown to improve accuracy and the ability to generalize as well as reducing training time and the quantity of data needed (Khan, Hon et al. 2019). Its effectiveness in ecological camera trap applications has been established by (Norouzzadeh, Nguyen et al. 2017) and (Willi, Pitman et al. 2018).

3. Image Classification vs. Object Detection

The majority of camera trap image processing solutions achieve image classification rather than object detection (Gomez Villa, Salazar et al. 2016, Nguyen, Maclagan et al. 2017, Norouzzadeh, Nguyen et al. 2017, Willi, Pitman et al. 2018, Miao, Gaynor et al. 2019, Tabak, Norouzzadeh et al. 2019). Image classification is a process by which

a whole image is labeled as containing a given object, for example, if a pig is featured in an image, it will be labelled ‘pig. However, image classification is limited in situations where an image contains more than one species, e.g. a pig and a wildebeest (Schneider, Taylor et al. 2018). Object localization and counting is also not effectively achieved by image classification and models tend to struggle to distinguish between an empty frame and a small background object (Yousif, Yuan et al. 2019). In contrast, object detection is the process of locating and identifying one or more objects in an image. The model plots bounding boxes of varying classification confidence and association class labels, around each object in an image (see Figure 1 for comparison). It is more useful than image classification because it allows more information to be extracted from the images, such as the number of objects in an image, as well as information about reproduction, distribution, quantification and comparison of behavior across individual animals within a species group based on factors such as age and gender (Schneider, Taylor et al. 2018).

Another major benefit of object detection is the reduced impact of background and environmental features on object classification. Unlike image classifiers, which learn patterns in the entire image, object detectors only learn patterns within the constraints of the bounding boxes, and actively negative sample on the image background (area not included in the bounding boxes) (Wang, Hu et al. 2019, Zhao, Zheng et al. 2019). This enables object detectors to better generalize to new domains, thus facilitating location invariance. Despite these benefits, object detection necessitates a significantly higher expenditure of time and resources, due to the need to annotate all training images with bounding boxes and labels.

Consequently, most studies achieve image classification rather than object detection. In contrast, due to the major benefits provided by object detectors for automated camera trap image processing , this study focuses on object detection rather than image classification.

Several studies have achieved object detection in the context of camera trap image processing, however none have achieved location invariance, with testing using restricted to in-sample datasets. (Yousif, Yuan et al. 2019) employed sequence-level background subtraction using handcrafted Histogram of Oriented Gradient (HOG) (Dalal and Triggs 2005) features to localize moving objects in camera trap images. This study did not aim to identify animal species, instead simply distinguished between humans and animals, and eliminated empty frames. Although it achieved high accuracy in this task, its application was not extended beyond eastern North America.

A novel ecological image processing software solution for use on a laptop by field ecologists and wildlife managers was developed by (Falzon, Lawson et al. 2020). It provides object detection and localization as well as species classification and object counting capabilities via training of YOLOv2 DarkNet-19 (Redmon and Farhadi 2016) Deep Convolutional Neural Networks (DCNN) on both daytime and infrared imagery. It boasts fast processing speeds and acceptable accuracy, achieved on a local machine, within a dedicated on-demand application. Tailored models can be applied to trap sites in Australia, New Zealand, North America, Serengeti and the USA. However, optimal performance is only achieved when models are trained and developed for a specific environment, camera trap imaging configuration and

species cohort. Thus, it suffers from lack of location invariance and robustness, as its accuracy and recall decrease significantly when it is used outside the scope of the environments on which it was trained.

(Schneider, Taylor et al. 2018) addressed the problem of object detection in camera trap images, with the aim of identifying, quantifying and localizing animal species. They used transfer learning to train a YOLOv2 model, achieving recall of 93% and accuracy of 80.4% on the Reconyx (www.reconyx.com) and Snapshot Serengeti (Swanson, Kosmala et al. 2015) datasets. The Reconyx dataset contained 946 images of 20 species, while the Snapshot Serengeti dataset contained 4,097 images of 48 species. They also trained a Faster R-CNN model (Ren, He et al. 2015) achieving 76.7% recall and 72.2% accuracy. They used a model pretrained on the MS COCO dataset (Lin, Maire et al. 2014) to initialize transfer learning. However, the robustness of the model was not evaluated on out of sample images, which is camera trap imagery obtained from traps and geographical locations not included in the training data. It also suffered from class imbalance with lower accuracy and recall for classes with fewer instances. Our research indicates this limitation can be overcome by sourcing images from publicly available data sources.

4. Improving Location Invariance via Dataset Construction

The suboptimal performance and inability of neural networks to generalize to contexts beyond the domain of the training data is a strong area of research interest. As early as 2008, studies in contextual object detection examined the consequences of 'unintentional regularities' in datasets resulting in object detectors learning associations between objects and their backgrounds, inhibiting their ability to detect

objects out of context (Hoiem, Efros et al. 2008, Sudderth, Torralba et al. 2008). (Everingham, Van Gool et al. 2010) noted that classifiers tend to learn the context of an object rather than model the appearance of the object. Thus, when the object is dissociated with its context, the classifier fails to detect it due to extensive use of image composition and context, resulting in a significant drop in performance. These findings were confirmed by (Miao, Gaynor et al. 2019) in an ecological context via the use of GRAD-CAM technology applied to models trained solely on camera trap images, illustrating the tendency of neural networks to learn background features as elements of an object if image background and context is not highly varied. It is therefore essential to broaden the context of animal imagery to extend beyond a restricted range of camera traps to ensure robustness and location and context invariance.

This phenomena of contextual association was also found by (Everingham, Van Gool et al. 2010) to be particularly prevalent in neural networks trained on images taken by researchers for a specific purpose. Consistencies within datasets, such as camera trap images collected within the context of a specific project, create an inner dataset bias, which results in the development of models less capable of generalization to other camera trap contexts. On this basis, we postulate that collection of camera trap images for neural network training mimics collection of images under laboratory or controlled conditions, whereby features such as lighting, camera angle, distance of objects from the camera, and background features are consistent across many images, thus encouraging contextual association. This is supported by (Willi, Pitman et al. 2018) who noted that their models, trained on camera trap images, would need to be retrained for use out of sample in other camera traps which did not form part

of the training set. In contrast, networks trained on data sourced from consumer photo sharing websites such as FlickR are more capable of generalization (Torralba and Efros 2011) due to the inherently high intra-dataset variability and reduced likelihood of inner dataset bias.

5. Review of Image Classification Solutions

(Gomez Villa, Salazar, & Vargas-Bonilla, 2016) proposed a method to automate species identification in a simplified version of the Snapshot Serengeti dataset, aiming to reduce the time and resource expenditure dedicated to analysis of large volumes of camera trap images. They experimented on six state of the art DCNNs, achieving accuracies ranging from 35.4% to 88.9% accuracy in the Top-1 classification task. Higher accuracies were only achieved when a balanced dataset containing only manually segmented foreground animals was used. This work was built upon by (Norouzzadeh et al., 2017) which used transfer learning to address factors such as the number of species in a given image, the presence of young, and animal behaviour. They achieved superior results (93.8%) on the Snapshot Serengeti dataset, but did not report results on out of sample data, meaning the robustness of the model was not evaluated. The transferability of the model development procedure is also limited by dependency on large datasets, which is not realistic in most practical ecology applications. Similarly, (Nguyen et al., 2017) performed species classification, comparing the performance of transfer learning and training from scratch. They used a subset of 107,022 images of 15 species from the Wildlife Spotter dataset (www.ala.org.au). They achieved AP results ranging from 84.39-96%. However, like (Norouzzadeh et al., 2017), they did not evaluate their model on an out of sample set, therefore its robustness cannot be assessed.

(Willi et al., 2018) attempted to address this issue by investigating the use of transfer learning to classify images from smaller camera trap datasets. They trained a model from scratch using the Snapshot Serengeti dataset, and then used transfer learning to repurpose the learned features for the task of image classification on the smaller trap datasets. They demonstrated that transfer learning does result in improved accuracy, however, this process is limited in value, as their model could not generalise well to other camera trap datasets. The model may have learned specific features and species biases within their training set. They noted that their model could only be used to classify images from the traps used for training and would need to be retrained for use in other camera traps characterised by differences in vegetation, camera placement, study sites and species. This limitation in model robustness and invariance to location and species variations is a significant factor inhibiting the deployment of DCNN models in camera trap applications. To address this, we propose the use of FlickR images to provide variety in background and image context to broaden the useability of trained models, thus shifting emphasis from quantity of data to quality of data. Furthermore, both (Norouzzadeh et al., 2017) and (Willi et al., 2018) developed deep learning solutions aimed for use alongside citizen science. In contrast, this study proposes a fully automated deep learning system, without reliance on citizen science.

To develop an image classification solution that could be used across all camera trap sites, (Tabak et al., 2019) trained a ResNet model on 3,741,656 images including 27 species from 5 locations in the US. They achieved 97.6% accuracy on test images from these sites. However, despite using data augmentation techniques such as

cropping, flipping, translation and brightness changes, their model only achieve 82% on out of sample test images. They acknowledged that the model was not location or background invariant and proposed training on all possible environments would be needed to increase accuracy. In an attempt to identify the underlying cause of location invariance (Miao et al., 2019) used gradient-weighted class-activation mapping (Grad-CAM) to illustrate the most salient features used by the network to classify species. This investigation provided evidence that DCNNs learn to associate the presence of environmental factors, such as trees, with particular species if they appear frequently in the training dataset. This inner dataset bias degrades the ability of the network to generalise to other datasets.

Reference List

Gomez Villa, A., Salazar, A., & Vargas-Bonilla, J. (2016). Towards Automatic Wild Animal Monitoring: Identification of Animal Species in Camera-trap Images using Very Deep Convolutional Neural Networks. *Ecological Informatics, 41*. doi:10.1016/j.ecoinf.2017.07.004

Miao, Z., Gaynor, K., Wang, J., Liu, Z., Muellerklein, O., Norouzzadeh, M. S., . . . Getz, W. (2019). Insights and approaches using deep learning to classify wildlife. *Scientific Reports, 9*. doi:10.1038/s41598-019-44565-w

Nguyen, H., Maclagan, S., Nguyen, T., Nguyen, T., Flemons, P., Andrews, K., . . . Phung, D. (2017). Animal Recognition and Identification with Deep Convolutional Neural Networks for Automated Wildlife Monitoring. doi:10.1109/DSAA.2017.31

Norouzzadeh, M. S., Nguyen, A., Kosmala, M., Swanson, A., Packer, C., & Clune, J. (2017). Automatically identifying wild animals in camera trap images with deep learning. *Proceedings of the National Academy of Sciences, 115*. doi:10.1073/pnas.1719367115

Tabak, M., Norouzzadeh, M. S., Sweeney, S., Vercauteren, K., Snow, N., Halseth, J., . . . Schlichting, P. (2019). Machine learning to classify animal species in camera trap images: Applications in ecology. *Methods in Ecology and Evolution, 10*, 585-590. doi:10.1111/2041-210X.13120

Willi, M., Pitman, R., Cardoso, A., Locke, C., Swanson, A., Boyer, A., . . . Fortson, L. (2018). Identifying Animal Species in Camera Trap Images using Deep Learning and Citizen Science. *Methods in Ecology and Evolution, 10*. doi:10.1111/2041-210x.13099
