# Supplementary material for: Automated location invariant animal detection in camera trap images using publicly available data sources
Source: Ecol Evol. 2021 Mar 10;11(9):4494–506. doi: 10.1002/ece3.7344 (PMC8093655; doi:10.1002/ece3.7344)
Supplement: Supplementary file 2 — Appendix S2 [file ECE3-11-4494-s001.pdf]

APPENDIX S2

Negative Sampling

The presence of negative samples in the training dataset is an essential component of any training pipeline (Ren, He et al. 2015). This is particularly important in single-class object detection (Gao, He et al. 2019) as it prevents over-enthusiastic classification of objects by allowing the network to discriminate between positive and negative samples. During multi-class object training, class characteristics are automatically differentiated during training. In contrast, the absence of alternative classes in single class object detection encourages the network to classify indiscriminately.

Thus, it is necessary to train DCNNs to recognize the distinctive features of pigs by training it to recognize which features are not attributable to pigs. For example, wildebeest and bison share very similar features, and inhabit similar ecosystems to warthogs. Therefore, it is extremely important to ensure a significant set of negative samples containing objects with similar characteristics to the chosen class is used during training. To rectify this problem, we downloaded 800 images of 38 animal species not included within our list of target species, including humans, as illustrated by Table 1. These images were included in all training sets for all single class trained models in this study.

Table 1: *Number of negative samples according to species type*

Species	Nº images	Species	Nº images
1. Chimpanzee	30	20. Elephant	14
2. Meerkat	31	21. Buffalo	17
3. Antelope	32	22. Wildebeest	15
4. Lion	16	23. Rat	13
5. Hippopotamus	30	24. Stumps	16
6. Chipmunk	30	25. Stones	15
7. Goat	33	26. Car	16
8. Rabbit	15	27. Bird	16
9. Bison	35	28. Turtle	14
10. Grizzly Bear	36	29. Cat	14
11. Kangaroo	33	30. Dingo	15
12. Wallaby	33	31. Dog	16
13. Boulders	9	32. Sheep	16
14. Raccoon	16	33. Horse	15
15. Tiger	14	34. Bear	30
16. Zebra	16	35. Giraffe	16
17. Human	119	36. Gorilla	16
18. Moose	16	37. Polar Bear	14
19. Spotted Hyena	40	38. Hare	16
**800 negative samples**			

The use of negative sampling incidentally solved another problem faced by ecologists. Differentiating between empty frames, and those containing a species of interest is often approached as a separate task to object detection. For example, (Willi, Pitman et al. 2018) trained two separate models; one was used to sort empty frames from those containing species of interest, and another to classify

species. This resulted in a positive bias in the species trained model, which led to misclassification. In contrast, we trained our models to automatically differentiate empty frames via inclusion of explicit negative sampling in the training set.

During preliminary experimentation, we found that due to the limited presence of non-target species within the FlickR and camera trap datasets, the trained models over enthusiastically classified any animal-like or foreground object as belonging to a target class. Our experimental results strongly indicate that without a negative sample dataset, models will classify instances of non-target species as target species. For example, without negative sampling, the bear and dog in Figure 1 were classified as the species 'pig' with relatively high confidence.

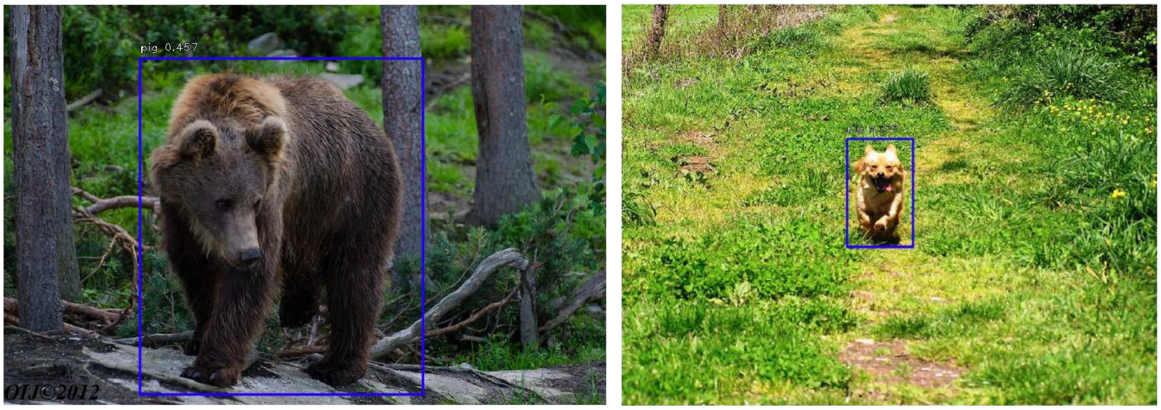

Figure 1: *Without explicit negative sampling, the network classified the bear on the left as a pig, with 45.7% confidence. Similarly, it classified the dog on the right as a pig, with 77.7% confidence.*

Experimental results suggest that the ratio of negative samples to positive samples should not be below 2:1. An interesting area of future research would be to determine the optimum ratio of negative to positive samples in single class object detection. Another potential area of research would be to examine whether explicit negative sampling is beneficial in multi-class object detection.

Furthermore, each test set used in this study was supplemented by 200 empty images, or images containing non-target species, to prevent biased evaluation of false positives. These were randomly selected from the same dataset from which the training images were obtained. For example, 200 images from Snapshot Serengeti, not containing striped hyenas, but including images of spotted hyenas, amongst other species, and empty images, were added to the Snapshot Serengeti test set used across all striped hyena experiments.

Reference List

Gao, L., Y. He, X. Sun and X. Jia (2019). "Incorporating Negative Sample Training for Ship Detection Based on Deep Learning." Sensors **19**: 684.

Ren, S., K. He, R. Girshick and J. Sun (2015). "Faster R-CNN: Towards Real-Time Object Detection with Region Proposal Networks." IEEE Transactions on Pattern Analysis and Machine Intelligence **39**.

Willi, M., R. Pitman, A. Cardoso, C. Locke, A. Swanson, A. Boyer, M. Veldthuis and L. Fortson (2018). "Identifying Animal Species in Camera Trap Images using Deep Learning and Citizen Science." Methods in Ecology and Evolution **10**.
