# Supplementary material for: Automated location invariant animal detection in camera trap images using publicly available data sources
Source: Ecol Evol. 2021 Mar 10;11(9):4494–506. doi: 10.1002/ece3.7344 (PMC8093655; doi:10.1002/ece3.7344)
Supplement: Supplementary file 4 — Appendix S4 [file ECE3-11-4494-s003.pdf]

APPENDIX S4: SSIM Image Duplicate Remover

Andrew Shepley

I. Algorithm Pseudocode

Algorithm 1 SSIM - Duplicate Remover

Input: $dir1$, $dir2$, $dir3$
$dir1$ is the directory containing all images
$dir2$ is an empty directory to contain unique images
$dir3$ is an empty directory to contain duplicate images
begin:
for f_i in $dir1$ **do**
  $imageA \leftarrow f_i$
  while $dir1$ contains more than one file **do**
    if $Algorithm2(dir1, imageA)$ is $True$ **then**
      $dir3 \leftarrow dir3 \cup f_i$
    else
      $dir2 \leftarrow dir2 \cup f_i$
    end if
    $dir1 \leftarrow dir1 - f_i$
  end while
  if $length(dir1) == 1$ **then**
    if $Algorithm2(dir2, imageA)$ is $True$ **then**
      $dir3 \leftarrow dir3 \cup f_i$
    else
      $dir2 \leftarrow dir2 \cup f_i$
    end if
    $dir1 \leftarrow dir1 - f_i$
  end if
end for
return $dir2$

Algorithm 2 SSIM - Duplicate Remover

Input: dir, $imageA$
$dir1$ is the directory containing all images
$image$ is an image to compare to all images in the directory
begin:
for f_i in dir **do**
  if f_i != $imageA$ **then**
    $imageB \leftarrow f_i$
    $score \leftarrow compareSSIM(imageA, imageB)$
    if $score == 1$ **then**
      return $True$
    end if
  end if
end for
return $False$
