# Supplementary material for: Automated location invariant animal detection in camera trap images using publicly available data sources
Source: Ecol Evol. 2021 Mar 10;11(9):4494–506. doi: 10.1002/ece3.7344 (PMC8093655; doi:10.1002/ece3.7344)
Supplement: Supplementary file 5 — Appendix S5 [file ECE3-11-4494-s007.pdf]

APPENDIX S5

Application to Real Life Multi-Class Detection Problem

To demonstrate the application of this training method to multi-class tasks, we trained a RetinaNet model (*FlickR_multiclass*) according to the specifications provided in Appendix S3. We downloaded 300 images of each of the classes pig, kangaroo, fox and goat from FlickR, along with approx. 1000 negative samples. Search queries for both positive and negative samples are provided in Table 1.

Table 1: *Search queries used to download images from FlickR. For search queries related to the class 'pig', see Table 1 in the main study.*

Kangaroo	Goat	Fox
macropus AND giganteus<br>*macropus AND fuliginosus*<br>*eastern AND grey AND kangaroo*<br>*infrared OR jumping AND kangaroo OR kangaroos*<br>*kangaroo AND eating*	*capra AND hircus*<br>*feral AND goat OR goats*<br>*wild AND goat OR goats*<br>*Bouc OR chevre*	*vulpes*<br>*fox or Foxes*<br>*renard OR renards*<br>*Vulpes vulpes*<br>*Australian OR Australia AND fox*<br>*Fox OR foxes AND infrared*

The model was then tested on an out of sample trap site dataset (Scotts, NSW) with class distribution illustrated by Figure 1. Note that classes with less than 10 instances were ignored for practical purposes.

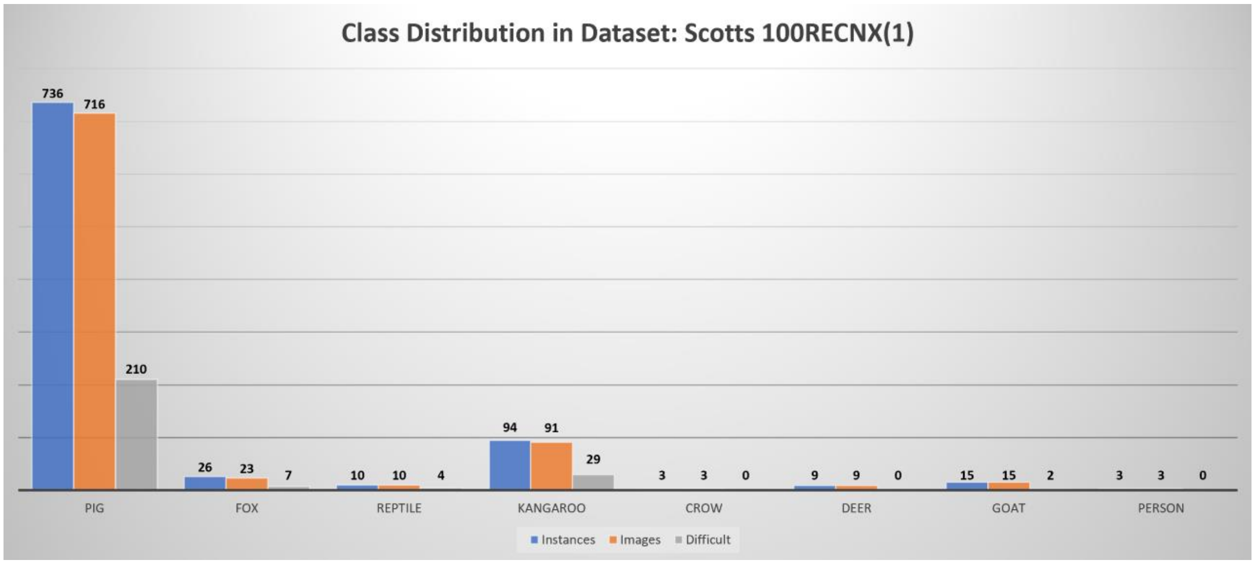

Figure 1: *Class distribution of out of sample Scotts multi-class test set. The class pig was the most commonly observed class. All classes with 10 or less instances were ignored. This dataset is available to the git repository associated with this study.*

Results obtained using the *FlickR_multiclass* model are presented in Table 1. The strongest performing class was 'pig' with a mAP of 90.87%. The weakest performing class was 'fox' with a mAP of 23.76%.

CLASS	MAP (%)
Pig	90.87
Kangaroo	66.83
Goat	66.34
Fox	23.76
**mAP (%)**	61.95

Table 1: *mAP results obtained by FlickR_multiclass (without infusion).*

To improve the results, we infused the FlickR training data with out of sample trap images from another 2 sites (Kilparney and Yarra, both locations in Northern NSW, Australia). We refer to the infusion model as *inf_multiclass_10.* We used 10% infusion as it was found to achieve good results in the main study. This meant only 30 images from each class was required (120 images of 4 species).

The results of obtained by *inf_multiclass_10* on the Scotts test dataset are presented in Table 2. The overall mAP increased by 18.99%. The fox class mAP improved the most, with an increase of 42.76%. The mAP improved across all classes.

CLASS	AP (%)
Pig	94.61
Kangaroo	76.85
Goat	85.77
Fox	66.52
**mAP (%)**	80.94

Table 2: *mAP results obtained by inf_multiclass_10 on the out of sample Scotts test set.*

Sample images are provided in Figures 2 and 3. These illustrate correctly classified animals as well as false positives. Image results obtained by *inf_multiclass_10*.

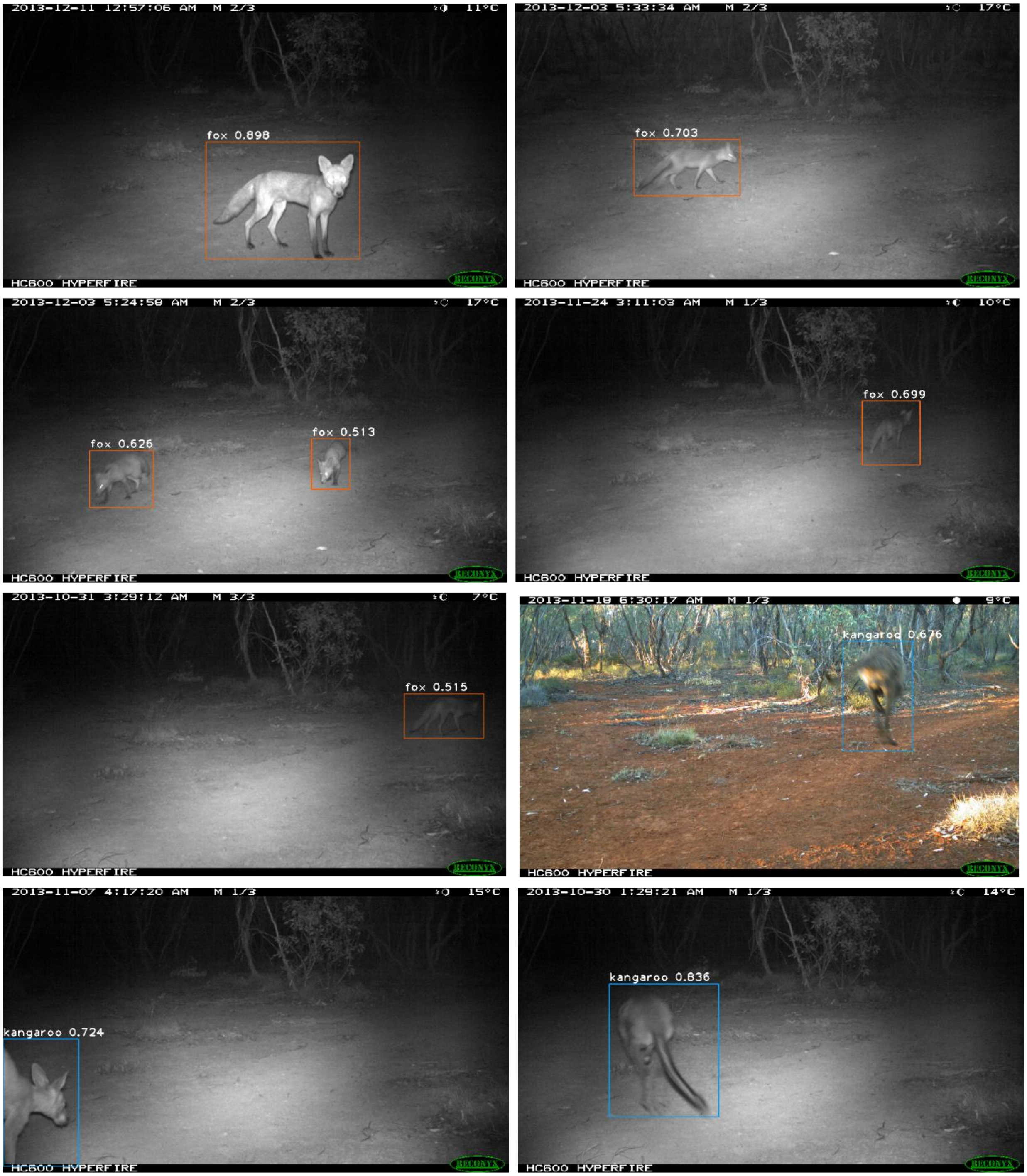
2013-12-11 12:57:06 AM M 2/3 11°C
fox 0.898
HC600 HYPERFIRE
2013-12-03 5:33:34 AM M 2/3 17°C
fox 0.703
HC600 HYPERFIRE
2013-12-03 5:24:58 AM M 2/3 17°C
fox 0.626
fox 0.513
HC600 HYPERFIRE
2013-11-24 3:11:03 AM M 1/3 10°C
fox 0.699
HC600 HYPERFIRE
2013-10-31 3:29:12 AM M 3/3 7°C
fox 0.515
HC600 HYPERFIRE
2013-11-18 6:30:17 AM M 1/3 9°C
kangaroo 0.676
HC600 HYPERFIRE
2013-11-07 4:17:20 AM M 1/3 15°C
kangaroo 0.724
HC600 HYPERFIRE
2013-10-30 1:29:21 AM M 1/3 14°C
kangaroo 0.836
HC600 HYPERFIRE

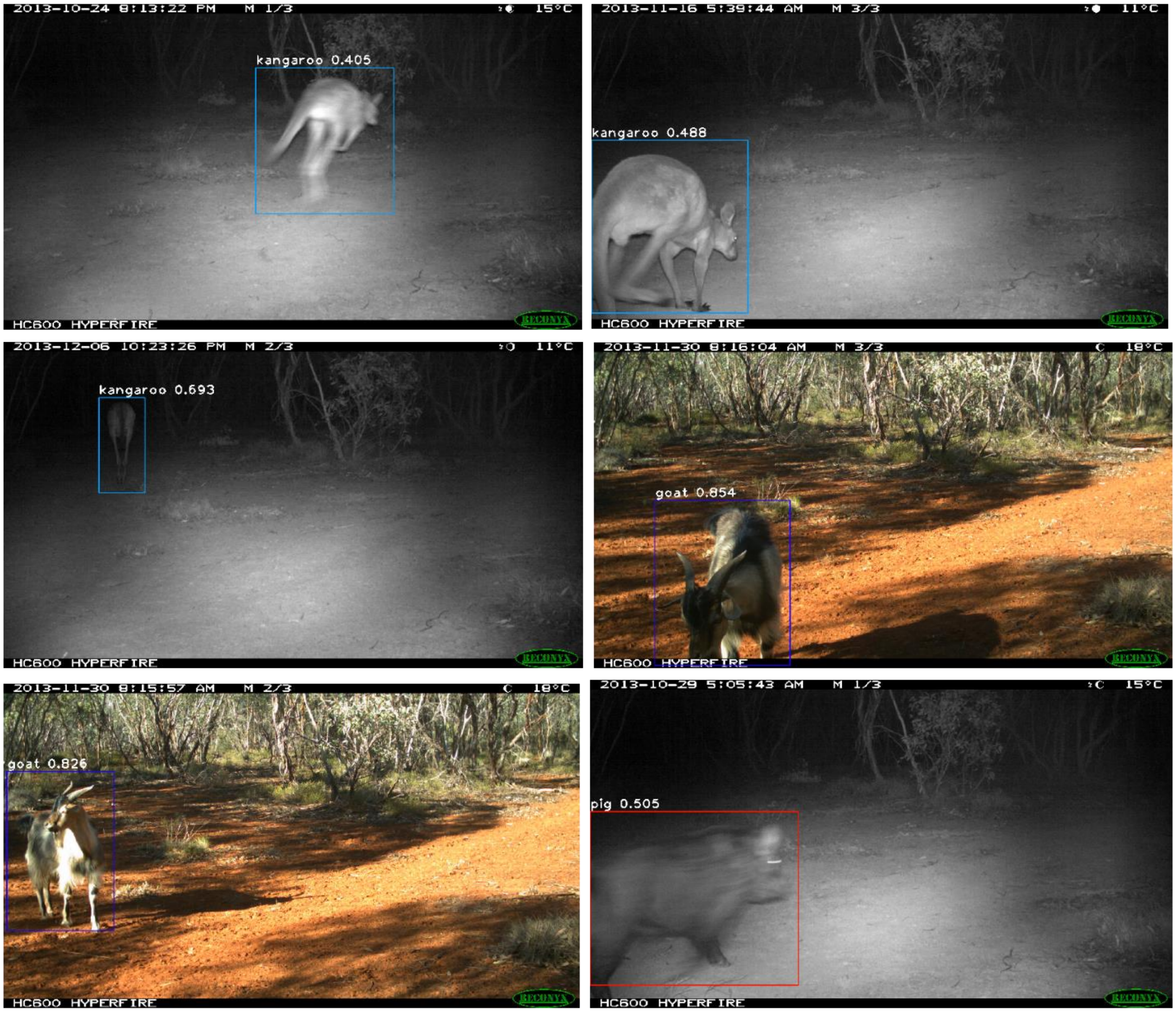

2013-10-24 8:13:22 PM M 1/3 15°C
kangaroo 0.405
HC600 HYPERFIRE
2013-11-16 5:39:44 AM M 3/3 11°C
kangaroo 0.488
HC600 HYPERFIRE
2013-12-06 10:23:26 PM M 2/3 11°C
kangaroo 0.693
HC600 HYPERFIRE
2013-11-30 8:16:04 AM M 3/3 18°C
goat 0.854
HC600 HYPERFIRE
2013-11-30 8:15:57 AM M 2/3 18°C
goat 0.826
HC600 HYPERFIRE
2013-10-29 5:05:43 AM M 1/3 15°C
pig 0.505
HC600 HYPERFIRE

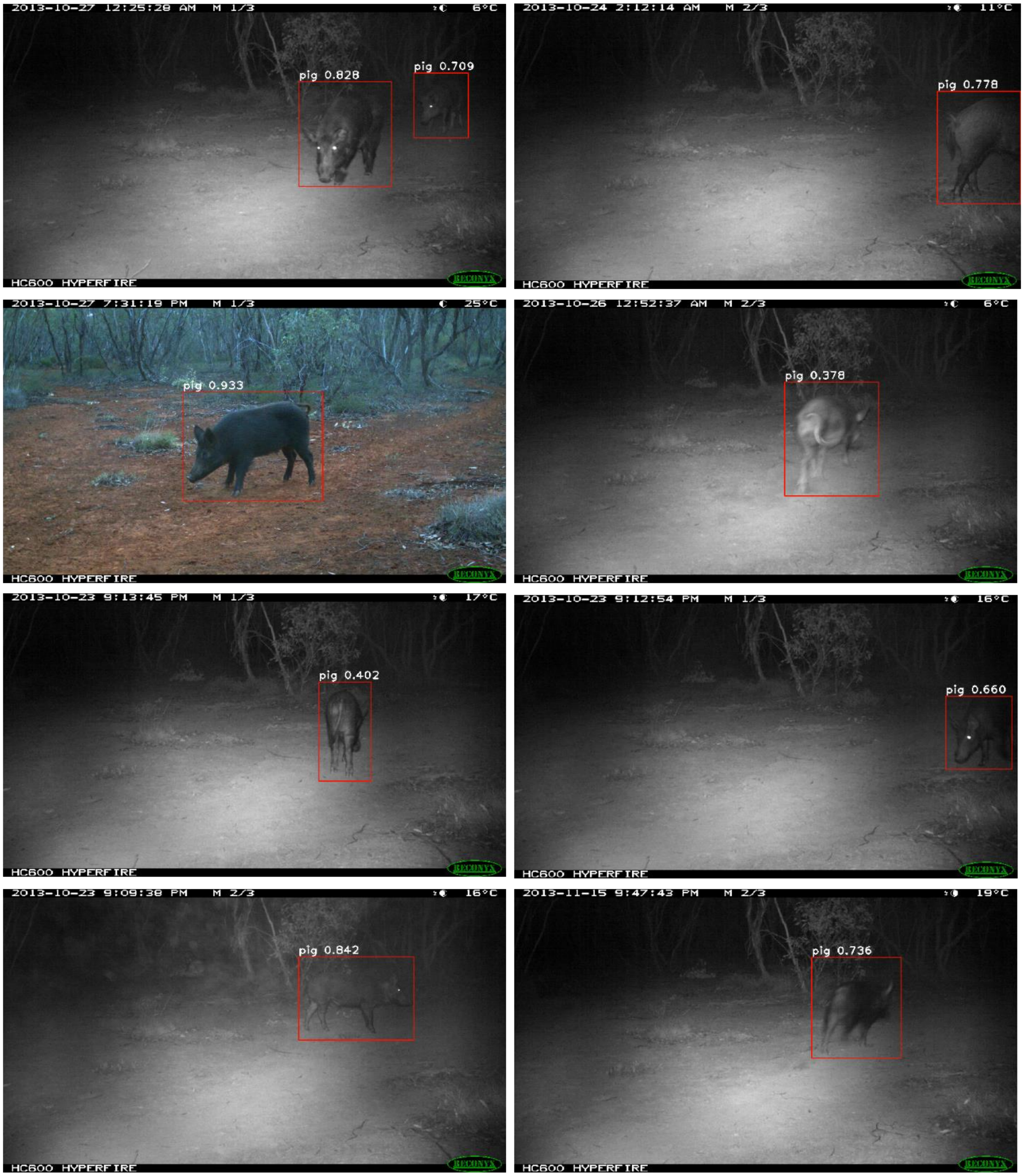

Figure 2: *Correctly classified and located classes.*

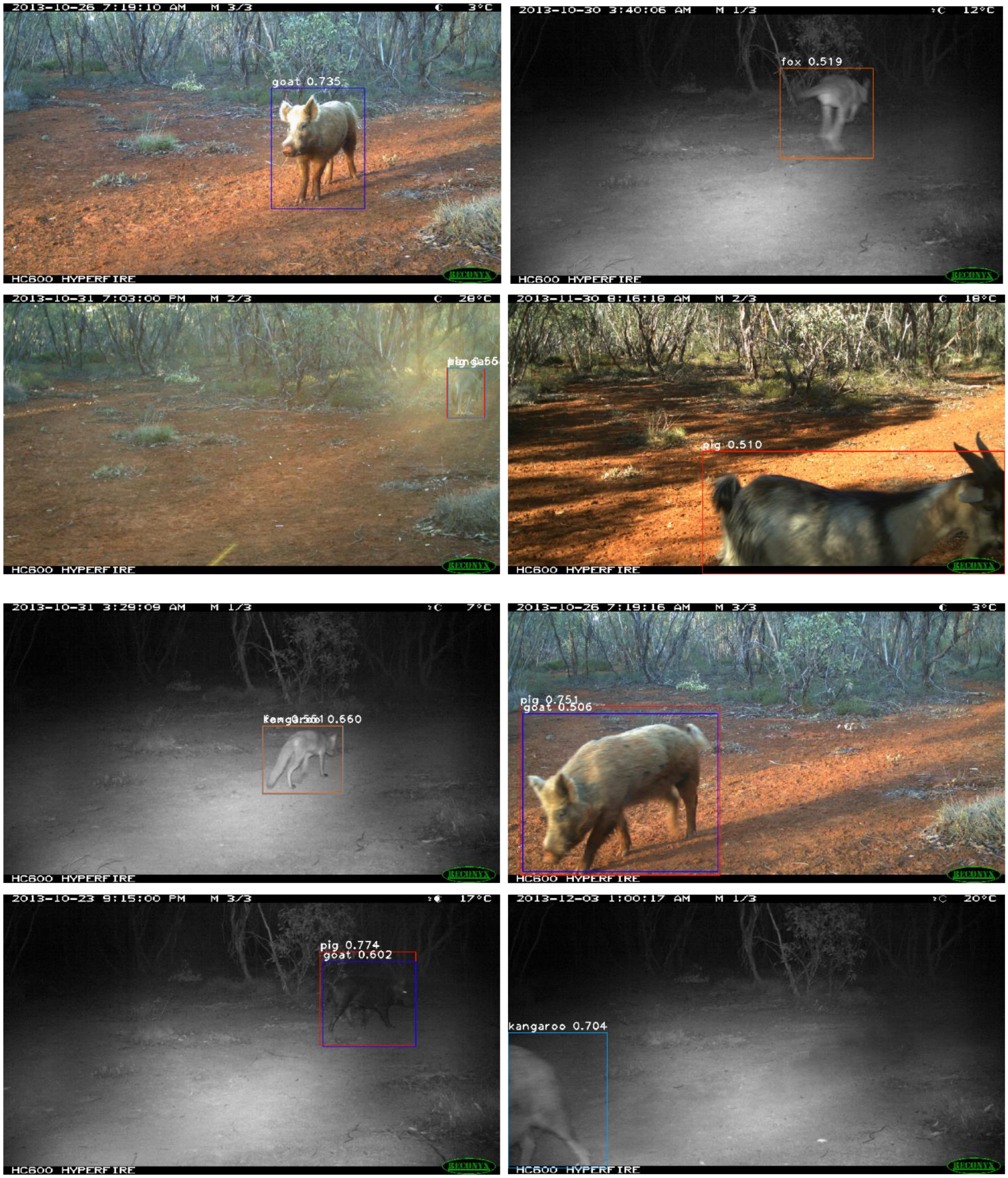

Figure 3: *Incorrectly classified classes.*
