# Supplementary material for: Automated location invariant animal detection in camera trap images using publicly available data sources
Source: Ecol Evol. 2021 Mar 10;11(9):4494–506. doi: 10.1002/ece3.7344 (PMC8093655; doi:10.1002/ece3.7344)
Supplement: Supplementary file 6 — Appendix S6 [file ECE3-11-4494-s002.pdf]

APPENDIX S6

In Sample Infusion

Methodology

We conducted further experiments to ascertain the effect of a small subsample of in-sample images. This would allow ecologists to improve the performance of their location invariant model with a minimal infusion of their own project-specific camera trap images. Due to limited availability of camera trap data for the rhinoceros and striped hyena classes, we conducted the in-sample experiments only on the pig class. Four models were trained for each pig location, namely, AU_pig, SS_pig, CC_pig, NA_pig, and EU_pig. Just like the out of sample infusion experiments presented in the main study, incremental infusion of camera trap imagery into training datasets was conducted to observe the impact of the introduction of trap images into the FiN training. Subsets of images from each camera trap training set were added in increments of 5% as shown by Table 1.

Table 1: *Incremental infusion of camera trap images into FiN training. Each model is named according to the source of the infusion data, and the percentage infusion, e.g. SS_inf_10 is a model trained on FiN data, infused with 60 (10%) Snapshot Serengeti images. Each training set contains 800 negative samples. Each test set was supplemented with 200 negative samples.*

Location/dataset	Models	Infusion Images	Training sets	Test sets
**Tanzania (SS_pig)**	*SS_inf_05*	30	1424	397
	SS_inf_10	60	1454	
	SS_inf_15	90	1484	
	SS_inf_20	120	1514	
**North America (NA_pig)**	*NA_inf_05*	30	1424	343
	NA_inf_10	60	1454	
	NA_inf_15	90	1484	
	NA_inf_20	120	1514	
**South Africa (CC_pig)**	*CC_inf_05*	30	1424	383
	CC_inf_10	60	1454	
	CC_inf_15	90	1484	
	CC_inf_20	120	1514	
**Australia (AU_pig)**	*AU_inf_05*	30	1424	410
	AU_inf_10	60	1454	
	AU_inf_15	90	1484	
	AU_inf_20	120	1514	
**Europe (EU_pig)**	*EU_inf_05*	30	1424	331
	EU_inf_10	60	1454	
	EU_inf_15	90	1484	
	EU_inf_20	120	1514	

Each subset was added to the FiN/negative sample training set. Training was validated on a combination of the FlickR validation set and the camera trap validation sets. Testing was conducted on the unused camera trap images. For example, the Australian camera trap training set was comprised of 530 images. The largest infusion percentile used was 20% of the size of the FlickR

dataset, which means 120 images were used for infusion training. The remaining 410 images were used for testing.

Results

The results of the in-sample infusion experiments are presented in Figure 1. These results indicate that infusion of a small subset of in-sample camera tap images can significantly improve performance of a model for a particular site.

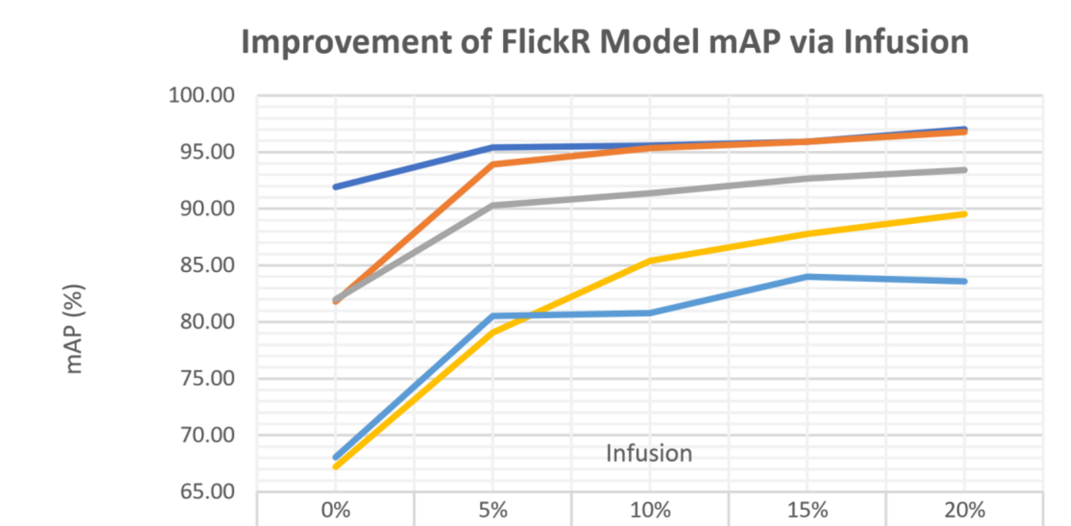

	0%	5%	10%	15%	20%
North America	91.92	95.40	95.58	95.93	97.02
South Africa	81.81	93.91	95.37	95.91	96.78
Tanzania	81.99	90.30	91.36	92.66	93.42
Europe	67.21	79.06	85.41	87.77	89.53
Australia	68.06	80.53	80.80	84.00	83.60

Figure 1: *The addition of 30-120 images (5-20%) obtained from the test site improved mAP results across models. From these results, we can recommend 10% in-sample infusion to achieve high accuracy location variant models, designed for site-specific optimized animal detection.*

In all cases, the infusion of 5-10% trap images into the FlickR training resulted in a significant increase in mAP (3.66-18.20%). Increasing the infusion set size from 10% to 20% did improve mAP results in 4 out of 5 cases, however improvements were not substantial (1.41-4.12%). Interestingly, the mAP of the Australian models began to decrease with the infusion of more than 15% trap images into training. We believe this is due to the poor image quality and lack of intra-dataset variability within the Australian image dataset.

In contrast, the mAP of the other infusion models continued to increase, albeit slightly. Notably, the models which benefited the most from infusion, were those infused with camera trap imagery containing infrared images, and/or the species *Phacochoerus africanus*. This may be a consequence of the relatively small percentage of *Phacochoerus africanus* in the FiN training set compared to *Sus*

scrofa. This suggests that it is of significant importance to achieve uniformity in subspecies distribution in the dataset to achieve an optimal mAP. Thus, class imbalance is not only detrimental across species (Willi, Pitman et al. 2018), but also appears to be within species variations. Another cause of the continued increase is the greater intra-dataset variability in the South Africa and Tanzania datasets – these sets contained images from more traps, with different backgrounds, camera angles, lighting etc. which made the addition of more images useful to the DCNN training set.

A qualitative example of the benefit of camera trap infusion into FiN training is provided by Figure 2, which illustrates the output of the FiN trained model, improvement with infusion of 5% camera trap images (AU_inf_05), and the output of alternative camera trap dataset training (South Africa, Tanzania, North America and Europe – see Appendix S6). Without the addition of camera trap images, the FiN model achieved mAP results of 68.06% on the Australian dataset, detecting all but the most difficult examples in the test set. With 5% infusion (30 trap images), the mAP increased by 12.47% to 80.53%. In contrast, the camera trap model trained on images from camera traps in North America, Europe, South Africa and Tanzania only achieved an AP of 68.96% on the Australian test set. These results suggest that FiN training with the addition of trap images in cases of underperformance is a more appropriate method of training DCNNs for camera trap image processing than camera trap training alone.

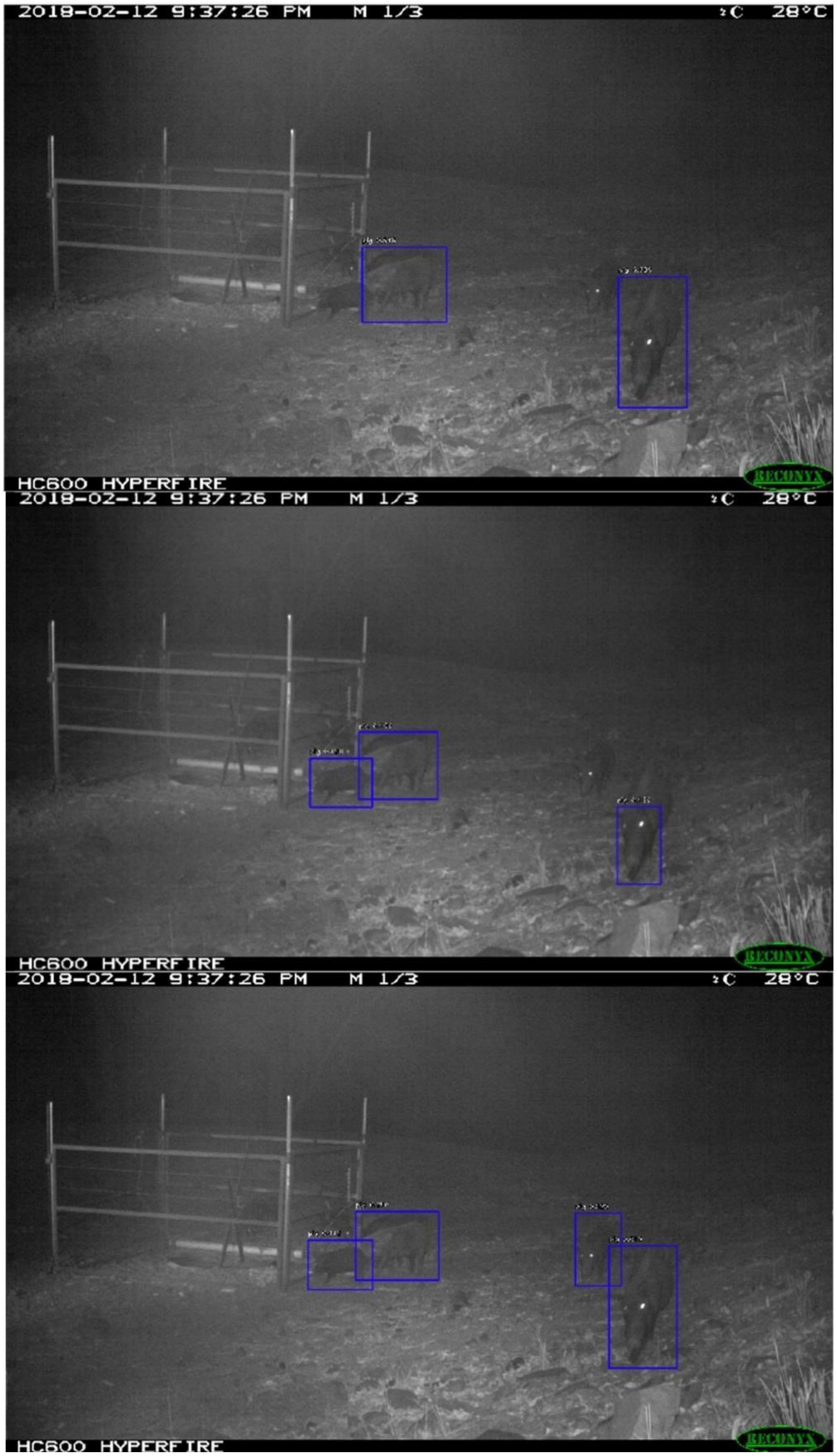

Figure 2: *Top: output from a model trained only on camera trap images (South Africa, Europe, North America and Tanzania) deployed on a test image from the AU_pig dataset. This model only detects 2 out of 5 pigs, whereas the FiN_pig model detects 3 out of 5 pigs (center). The AU_inf_05 model detects all 4 clearly visible pigs (bottom), only missing one highly occluded pig (far right).*

References

Willi, M., R. Pitman, A. Cardoso, C. Locke, A. Swanson, A. Boyer, M. Veldthuis and L. Fortson (2018). "Identifying Animal Species in Camera Trap Images using Deep Learning and Citizen Science." Methods in Ecology and Evolution **10**.
