# Supplementary material for: Automated location invariant animal detection in camera trap images using publicly available data sources
Source: Ecol Evol. 2021 Mar 10;11(9):4494–506. doi: 10.1002/ece3.7344 (PMC8093655; doi:10.1002/ece3.7344)
Supplement: Supplementary file 7 — Appendix S7 [file ECE3-11-4494-s006.pdf]

APPENDIX S7

Further Evaluation on Multiple Locations

To verify the method used in the main experiments on datasets/locations other than Snapshot Serengeti, we conducted further experiments. These experiments are conducted only on the class 'pig' due to greater availability in comparison to striped hyena and rhinoceros. The datasets used in this section are those described in Section 3(b) of the main study.

Methodology

We trained 4 single class RetinaNet models, namely AU_pig, NA_pig, EU_pig and CC_pig on the single location datasets bearing the same name. We used a 10% validation split. We then evaluated these models on the datasets they weren't trained on, to evaluate their ability to achieve location invariance. For example, the AU_pig model was trained on the AU_pig dataset, and was tested on the NA_pig, EU_pig, and CC_pig datasets. Details for each model are presented in Table 1.

Table 1: *Models trained on single location camera trap data, and evaluated on out of sample data from unseen locations.*

Model	Training set	Validation set	Test sets	Test set size
**AU_pig**	530	59	NA_pig<br>EU_pig<br>CC_pig	514<br>501<br>559
**NA_pig**	465	51	AU_pig<br>EU_pig<br>CC_pig	589<br>501<br>559
**EU_pig**	451	50	NA_pig<br>AU_pig<br>CC_pig	514<br>589<br>559
**CC_pig**	503	56	NA_pig<br>EU_pig<br>AU_pig	514<br>501<br>589

We also combined camera trap datasets from multiple locations and trained models on these combined datasets, to ascertain their performance in comparison to that of the FiN_pig model. Details are presented in Table 2.

Table 2: *Models trained on camera trap data from multiple locations and evaluated on out of sample camera trap images from unseen locations. Combined Trap Models are named according to the location acronyms of the datasets used for training, for example, CCNAEUSS stands for CC_pig, NA_pig, EU_pig, SS_pig.*

Combined Trap Models	Training Datasets	Training set size	Validation set size	Test dataset	Test set size
**CCNAEUSS**	CC_pig, NA_pig, EU_pig, SS_pig	2722	214	Australia (AU_pig)	589
**AUEUNASS**	AU_pig, EU_pig, NA_pig, SS_pig	2649	217	South Africa (CC_pig)	559
**AUNACCSS**	AU_pig, NA_pig, CC_pig, SS_pig	2801	223	Europe (EU_pig)	501
**AUCCEUSS**	AU_pig, CC_pig, EU_pig, SS_pig	2789	222	North America (NA_pig)	514

Results

The results of the multi-location experiments are presented in Figure 1. Interestingly, models trained on combined camera trap location data achieved competitive results, outperforming the FiN_pig model in 3 out of 4 cases. These results must be treated with caution however, as the FiN_pig model was only trained on 606 images, whereas the combined trap models were trained on 2649-2801 images. Therefore, the better performance may be attributable at least somewhat to the larger number of training images.

In the majority of cases, the models trained on camera trap data from a single location struggled to perform on out of sample images, with the worst model being CC_pig, which only achieved a mAP of 4.42% on the AU_pig dataset. The best single location model was EU_pig, which achieved a mAP of 63.78% on the North American dataset. This may be due to the higher similarity between background features present in this dataset.

In all cases, FiN_pig achieved good results on out of sample data in all 4 locations, with the lowest mAP being 67.21% on the Europe dataset, and the highest (91.92% mAP) being achieved on the NA_pig dataset. We believe these results demonstrate the general location invariance of FiN models and support the use of FiN data in the development of location invariant universal animal detectors.

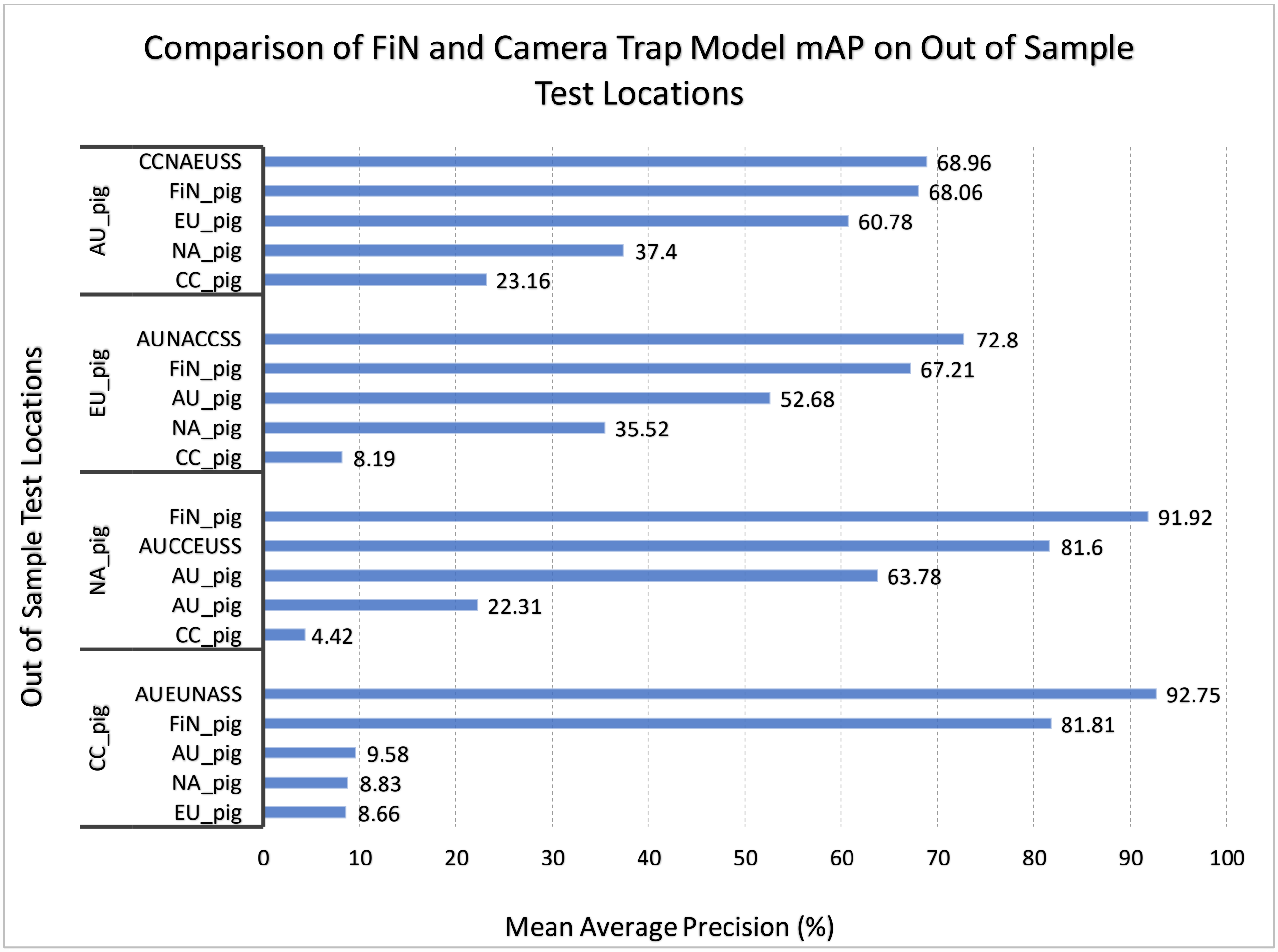

Figure 1: *Results of location invariance experiments for both single camera trap, and combined camera trap models, in comparison to FiN_pig.*
